# Physicochemical characterization and quantification of nanoplastics: applicability, limitations and complementarity of batch and fractionation methods

**DOI:** 10.1007/s00216-023-04689-5

**Published:** 2023-04-27

**Authors:** Maximilian J. Huber, Natalia P. Ivleva, Andy M. Booth, Irina Beer, Ivana Bianchi, Roland Drexel, Otmar Geiss, Dora Mehn, Florian Meier, Alicja Molska, Jeremie Parot, Lisbet Sørensen, Gabriele Vella, Adriele Prina-Mello, Robert Vogel, Fanny Caputo

**Affiliations:** 1 Institute of Water Chemistry (IWC), Chair of Analytical Chemistry and Water Chemistry, School of Natural Sciences (NAT, Dep. Chemistry), Technical University of Munich (TUM), Lichtenbergstr. 4, 85748 Garching, Germany; 2 Department of Climate and Environment, SINTEF Ocean AS, Trondheim, Norway; 3 Joint Research Centre (JRC), European Commission, Ispra, Italy; 4 Postnova Analytics GmbH, Landsberg am Lech, Germany; 5 Department of Biotechnology and Nanomedicine, SINTEF Industry, Trondheim, Norway; 6 Laboratory of Biological Characterization for Advanced Materials (LBCAM), Department of Clinical Medicine, Trinity Translational Medicine Institute, Trinity College Dublin, Dublin, Ireland; 7 School of Mathematics and Physics, The University of Queensland, St Lucia, QLD 4072 Australia; 8 Laboratoire National de Métrologie et d’Essais, Paris, France

**Keywords:** Analysis, Identification, Size, Morphology, Raman microspectroscopy, Pyrolysis gas chromatography mass spectrometry

## Abstract

A comprehensive physicochemical characterization of heterogeneous nanoplastic (NPL) samples remains an analytical challenge requiring a combination of orthogonal measurement techniques to improve the accuracy and robustness of the results. Here, batch methods, including dynamic light scattering (DLS), nanoparticle tracking analysis (NTA), tunable resistive pulse sensing (TRPS), transmission electron microscopy (TEM), and scanning electron microscopy (SEM), as well as separation/fractionation methods such as centrifugal liquid sedimentation (CLS) and field-flow fractionation (FFF)–multi-angle light scattering (MALS) combined with pyrolysis gas chromatography mass spectrometry (pyGC–MS) or Raman microspectroscopy (RM) were evaluated for NPL size, shape, and chemical composition measurements and for quantification. A set of representative/test particles of different chemical natures, including (i) polydisperse polyethylene (PE), (ii) (doped) polystyrene (PS) NPLs, (iii) titanium dioxide, and (iv) iron oxide nanoparticles (spherical and elongated), was used to assess the applicability and limitations of the selected methodologies. Particle sizes and number-based concentrations obtained by orthogonal batch methods (DLS, NTA, TRPS) were comparable for monodisperse spherical samples, while higher deviations were observed for polydisperse, agglomerated samples and for non-spherical particles, especially for light scattering methods. CLS and TRPS offer further insight with increased size resolution, while detailed morphological information can be derived by electron microscopy (EM)–based approaches. Combined techniques such as FFF coupled to MALS and RM can provide complementary information on physical and chemical properties by online measurements, while pyGC–MS analysis of FFF fractions can be used for the identification of polymer particles (vs. inorganic particles) and for their offline (semi)quantification. However, NPL analysis in complex samples will continue to present a serious challenge for the evaluated techniques without significant improvements in sample preparation.

**Graphical abstract:**

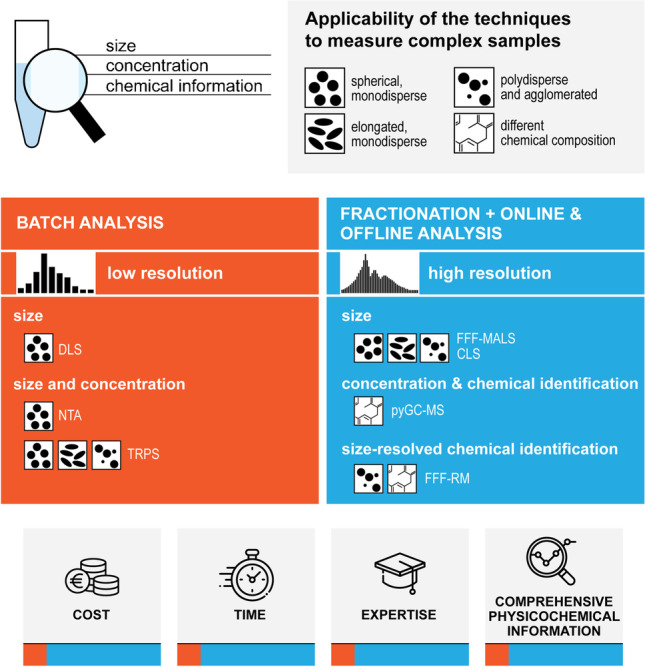

**Supplementary Information:**

The online version contains supplementary material available at 10.1007/s00216-023-04689-5.

## Introduction

The toxicological and environmental effects of nanoplastics (NPLs) are currently being heavily debated among scientists and across society [1–5]. NPLs are hypothesized to be a ubiquitous contaminant, derived from direct emissions or being generated through the degradation and fragmentation of larger plastic litter items [6]. The environmental fate, transport, and behavior of NPLs will be governed by their specific physicochemical properties, e.g., density, small size (< 1 µm), and high specific surface area. The combination of these properties, among others, distinguishes NPLs significantly from microplastics (1 µm–5 mm) and engineered nanomaterials (ENMs). Compared to that from ENMs, the potential global pollution from NPLs is much higher due to the much more widespread use of plastics [7]. However, virtually nothing is known about their actual levels in the environment and the potential risks associated with exposure to environmentally relevant NPLs [8]. Owing to the expected low mass-based concentration in environmental matrices, the very small particle size, and the need to identify and quantify NPLs in complex matrices [9–11], analytical methodologies are currently under development. As exposure assessment is a critical component in risk assessment, it is still not possible to fully assess the environmental or human health risks associated with NPLs [12, 13].

The few available studies have reported the presence of NPLs in surface water [14], alpine snow [15], and soil [16, 17]. From a hazard perspective, the adverse effects of NPLs are most likely not governed by a single attribute, but depend on particle size, shape, polymer type, and degradation/oxidation state, as well as the presence of chemical additives and sorbed substances, potentially all being interconnected factors influencing the toxicity. To enable both exposure assessment and risk assessment of NPLs, there is a need to improve existing methods for NPL identification and quantification, as well as for developing, validating, and standardizing new analytical approaches [18, 19]. To achieve this, representative test materials and, eventually, reference materials are urgently needed [20]. However, only a few studies have reported the production and use of more complex/realistic NPLs for toxicity studies on biota [21, 22]. Due to the lack of readily available, representative test materials that mimic the real NPL heterogeneity, as well as being fit for purpose for the complexity of the measured parameters, the accuracy and selectivity of analytical approaches can only be verified by demonstrating that the measured result is comparable to the measured result of a second, well-characterized analytical procedure (e.g., an orthogonal procedure) [23]. Furthermore, it is necessary to combine complementary analytical approaches for measuring different parameters that may impact the toxicity of NPLs, developing an integrated characterization strategy. In this context, it is important to conduct comparative studies to determine the advantages and limitations of different methods with respect to NPL analysis. For example, Caputo et al. used spherical monodisperse NPL particles for the comparison of different size characterization methods [24]. While this enables a very detailed performance comparison of fundamentally different techniques, as a next step, it is necessary to utilize test materials for further studies that are representative of the heterogeneity of NPL physicochemical properties thought to be present in the environment.

To obtain information on particle size, size distribution, and other physical parameters, several orthogonal techniques can be applied to NPL analysis [10, 24], each based on different measurement principles, resulting in differences in both their applicability and limitations, depending on the sample properties. Particle size, size distribution, morphology, and even the concentration of NPLs can be determined using a combination of light scattering–based methods (e.g., dynamic light scattering, DLS; nanoparticle tracking analysis, NTA; multi-angle light scattering, MALS), centrifugal liquid sedimentation (CLS) [25], imaging methods (e.g., transmission electron microscopy, TEM; scanning electron microscopy, SEM), and impedance methods (e.g., tunable resistive pulse sensing, TRPS). However, none of these methods is really able to identify the chemical nature of the measured particles nor distinguish plastic particles from non-plastic particles in a mixed sample. NPL-specific data can be only generated if a sample preparation methodology is able to isolate a pure NPL fraction from any other particulates, impurities, or matrix components that might be present in an environmental sample. Commonly used approaches for isolating microplastic samples from inorganic environmental matrices (particle size range  > 1 µm), such as density separation, are not practically applicable to NPLs. Furthermore, the most common methods used for the digestion of the (in)organic environmental matrices prior to microplastic fractionation from the matrix residues (e.g., enzyme, acid, base or oxidative digestion) have not been validated for NPLs [26–29]. Given their very high surface to volume ratios, NPLs could easily be destroyed or damaged by such processes.

In recent years, the use of hyphenated fractionation methods, which are applicable to other kinds of nanomaterials, has gained an increasing amount of attention for the identification and quantification of NPLs [30–32]. These methods can be used to fractionate and isolate the nanoparticles from the environmental matrices and then to measure a comprehensive set of physicochemical property and concentration data on fractions with a narrow size distribution in a comparatively short time, especially if online coupling of several detectors can be achieved. Critical to the approach is the isolation of specific particle size fractions within the nano-range (1–1000 nm). For example, field-flow fractionation (FFF) techniques can produce defined particle size fractions that can be used in hyphenation (online coupling) with multiple detectors, like MALS and Raman microspectroscopy (RM), to achieve size-resolved chemical information [30, 33]. In FFF, a thin ribbon-like channel is generally used for the separation. In asymmetrical flow FFF (AF4), the separation force is realized by applying a secondary flow perpendicular to the laminar channel flow, while for centrifugal FFF (CF3), a centrifugal force is utilized. MALS is a powerful technique to determine particle size (distribution). In combination with other methods, like online DLS, shape parameters can also be obtained [34]. In contrast, offline-coupled detectors can provide complementary information with higher resolution and sensitivity on fractionated samples, with the drawback of needing to perform multiple measurements. In the case of offline pyrolysis gas chromatography mass spectrometry (pyGC–MS), a selective mass concentration of NPLs can be obtained [33], which is currently not accessible for environmental NPL samples with online detectors.

In this study, we compare several methods, including batch techniques, e.g., DLS and NTA; single-particle-based approaches, e.g., TRPS, SEM, TEM, and CLS; and combined fractionation methods, e.g., FFF–MALS, pyGC–MS, and RM, for the analysis of NPLs and inorganic NPs using a broad range of representative test materials. To move a step closer toward mimicking real NPL physicochemical complexity, polystyrene (PS) and polyethylene (PE) NPLs of different sizes and with different degrees of agglomeration were synthesized and used as representative test samples. Complementary inorganic NPs were included in the study for multiple purposes. Monodisperse, spherical silica NPs were used as a quality control particle for sizing and concentration measurements. Spherical and elongated iron oxide NPs (FeOx) were used to evaluate the capability of sizing and concentration techniques to measure non-spherical constituent particles, prior to testing the selected techniques on more complex, non-spherical PS and PE agglomerates. Furthermore, titanium dioxide (TiO_2_) NPs were employed in mixtures of plastic and inorganic particles to test the capability of FFF–RM to perform size-resolved measurements and to distinguish different particles by their chemical composition.

The technique-related differences, including the applicability and limitations in terms of accuracy and selectivity of individual batch techniques, were evaluated by comparing the results obtained by measuring samples of increasing analytical complexity. This includes particle number concentrations measured by TRPS, NTA, and CLS; particle morphology determined by TEM and SEM; and information on mass concentration gained from pyGC–MS data. Finally, chemical information was determined by RM and pyGC–MS, with online and offline coupling to FFF, respectively. In the case of FFF–pyGC–MS, a suitable sample preparation approach and procedures for the FFF coupling have been developed and tested in two different laboratories. The study highlights the strengths of using combined techniques for a more comprehensive physicochemical characterization and quantification of NPLs.

## Methods and materials

### General sample preparation

An overview of (i) the PS and PE NPLs, (ii) the inorganic nanoparticle materials used in this study, and (iii) the characterization techniques selected for each sample is provided in Table 1. The test materials and samples were selected by considering the technical requirements and challenges associated with each analytical technique considered in the study. PS, PE, and FeOx materials were used for multiple assays, including batch sizing measurements by DLS, TRPS, NTA, and CLS; coupled methods including CF3–MALS (2 labs); and chemical analysis by RM in batch mode and online-coupled CF3–RM. Additionally, monodisperse silica nanoparticles of different sizes between 50 and 1000 nm were used as a quality control for the sizing techniques in the size range of interest, as described in the Supplementary material (SM, Table S1–S3). Poly(methyl methacrylate) (PMMA) NPLs and TiO_2_ NPs were tested during the development of online CF3–RM coupling to differentiate between inorganic and organic particles of the same particle size. Finally, multimodal mixtures of polymeric NPs were analyzed by combining AF4 fractionation and offline pyGC–MS analysis.

**Table 1:** List of samples and mixtures used in this study. Sample description, including the supplier, the expected particle concentration, the expected size, the expected particle shape, the measured particle density, and the chemical composition, and the list of the complementary techniques used in this work are reported for each sample. Particle concentration (particle mL^−1^) measurements for the stock solution are reported in Table 3

Sample	Supplier	Stock concentration (% mass)	Expected average size (shape)	Particle density (g mL^−1^)**	Chemical composition	Techniques used for analysis
PE1	SINTEF Industry, Norway	5%	400 nm (spherical)	1.002 (± 0.002)	Polyethylene (PE) particles in water stabilized with 0.5% Tween 80, polydisperse	DLS (2 labs), NTA, TRPS, TEM, RM, CF3–MALS (2 labs), CF3–RM
PS1	SINTEF Industry, Norway	10.3%	150 nm (spherical)	1.052 (± 0.001)	Polystyrene (PS) particles in water stabilized with 2 g L^−1^ NaLS, monodisperse	DLS (2 labs), NTA, TRPS, CLS, SEM, RM, CF3–MALS (2 labs)
PS2	SINTEF Industry, Norway	16%	200 nm (spherical)	1.051 (± 0.003)	PS particles doped with Eu, dispersed in water stabilized with 3 g L^−1^ NaLS, monodisperse	DLS (2 labs), NTA, CLS, SEM, TEM, RM, CF3–MALS (2 labs), CF3–RM, (AF4–)pyGC–MS
PS3	SINTEF Industry, Norway	16%	250 nm (spherical)	1.042 (± 0.002)	PS particles in water stabilized with 3 g L^−1^ NaLS, polydisperse	DLS (2 labs), NTA, TRPS, CLS, SEM, RM, CF3–MALS (2 labs), CF3–RM, (AF4–)pyGC–MS
FeOx100*	Cotton Mouton Diagnostics Ltd, UK	Not provided	100 nm (spherical)	3.710 (± 0.110)	Iron oxide nanospheres, monodisperse	DLS (2 labs), NTA, TRPS, CLS, SEM, TEM, RM, CF3–MALS
FeOx2000*	Cotton Mouton Diagnostics Ltd, UK	Not provided	410 × 80 nm (rod)	3.710 (± 0.080)	Iron oxide nanorods, monodisperse	DLS (2 labs), NTA, TRPS, CLS, SEM, TEM, RM, CF3–MALS
PSL60	Thermofisher Scientific 3060A	1%	60 nm (spherical)	n.d.	PS particles, monodisperse	(AF4–)pyGC–MS
PMMA500	microParticles GmbH, Germany	5%	500 nm (spherical)	n.d.	PMMA particles, monodisperse	(CF3–)RM
TO70	Evonik Industries AG, Germany	40%	70 nm (irregular)	n.d.	Titanium dioxide nanoparticles, polydisperse	(CF3–)RM
Mixture MS1	–		–		7.1 mg L^−1^ PS2, 34.7 mg L^−1^ PS3, 31.4 mg L^−1^ PSL60	(AF4–)pyGC–MS
Mixture RM1	–		–		5.0 mg L^−1^ PE1, 3.2 mg L^−1^ PS2, 0.1 mg L^−1^ PMMA500	(CF3–)RM
Mixture RM2	–		–		4.8 mg L^−1^ PS2, 0.1 mg L^−1^ PMMA500, 8.0 mg L^−1^ TO70	(CF3–)RM

*n.d.* not determined

*Mass concentration of the stock not provided, stocks 10 × , 10 times more concentrated than the usual stocks provided by the commercial provider

**Particle density measured by CF3–MALS: The particle density values were obtained from CF3 theory based on the observed retention time and the particle size obtained from MALS evaluations. The CF3 channel height was determined using fractionated PS size standards

Stock suspensions of the NPL and inorganic NP materials were first sonicated in a bath sonicator for 2 min and then diluted gravimetrically in a 0.0125% (v/v) solution of NovaChem100 (Postnova Analytics GmbH (PN), Germany) in ultrapure water. The diluted suspensions were dispersed in a bath sonicator for an additional 2 min for NPLs or 30 min for FeOx particles before measurements, where ice was periodically added to the bath sonicator to avoid heating. Before use, all buffers used for particle dispersion were filtered through 0.2-μm filters made of a polyethersulfone (PES) membrane. This sample protocol was followed for the majority of analysis techniques conducted within the study, and any exceptions/deviations are noted in the respective analysis technique descriptions below.

### Dynamic light scattering (DLS)

DLS is one of the most commonly used techniques to assess particle size distribution [35]. It relies on light scattering of suspended particles. As the total scattering intensity of all particles is observed, however, the resulting size resolution is low [24, 36]. Furthermore, the signal of larger particles can cover the signal of smaller ones leading to a skewed size distribution [35, 37]. Although DLS is widely applied, it is not advisable for use as the only sizing method for the complex analysis of NPLs due to the aforementioned limitations [24].

The hydrodynamic diameters of all samples were determined using a Zetasizer Nano S (Malvern Panalytical, UK). DLS measurements were conducted at a temperature of 25 °C at a backscattering angle of 173°. The hydrodynamic diameter (*z*-average) and the dispersity from cumulative analysis were obtained according to ISO 22412 [38]. The results represent averages from at least 6 consecutive measurements, together with the corresponding standard deviation measured in two different laboratories.

### Nanoparticle tracking analysis (NTA)

NTA tracks the Brownian motion of each particle individually by detecting the scattered light over time [39]. Particle diameters can be calculated using the Stokes–Einstein equation for known temperature and viscosity of the suspension [37]. Besides particle number concentration, size, and size distribution, further properties like refractive index can be derived [40, 41]. NTA only offers a reliable working range of up to 800 nm for NPLs [24]. In most instances, NTA offers this information without in-depth knowledge on the sample properties (e.g., compared to density for CLS). Furthermore, NTA is able to handle a fairly wide concentration range, typically from 10^6^ to 10^9^ particles mL^−1^ [42]. However, the size resolution is significantly lower, especially compared to the non-light-scattering techniques described below [24, 36].

Analyses were conducted using a NanoSight NS500 system (Malvern Panalytical, UK) in light scattering mode using the EUNCL PCC-023 validation protocol [43]. The system is equipped with a 405-nm laser and operated using NanoSight 3.2 software. NPL and NP samples were incrementally diluted, in line with the Malvern Panalytical optimization protocol and as presented in the EUNCL PCC-023 SOPs, to achieve an optimal concentration of 20–100 particles per field acquisition (camera field) at a final working volume of 1 mL. A total of six videos of 60 s were recorded for each sample, the detection threshold during analysis was selected to ensure that only distinct nano-objects were analyzed, and the value was kept constant during the recording.

### Tunable resistive pulse sensing (TRPS)

TRPS can be used to obtain size, size distribution, concentration, and zetapotential information on particles ranging from 40 nm to 20 µm [24, 44]. In contrast to other sizing methods, TRPS measures the resistance when a particle passes through a pore, where the measured resistance due to a particle translocation through the pore is proportional to particle volume, leading to increased measurement sensitivity and size resolution [45]. Thus, TRPS determines the physical radius of a particle and not the hydrodynamic radius. To determine particle number concentration a single- or multi-pressure (depending on pore size) calibration procedure is necessary. The linear dependence of particle rate and pressure is used to calculate the particle concentration of the sample [46, 47]. The main advantage of this method for NPL analysis is that it can cover a large size range (40 nm to 20 µm) at high resolution when using different pore sizes. However, different sample preparation procedures, pore sizes, and measurement conditions may be needed to accommodate this range [24].

Measurements were conducted using equipment (qNano and Exoid) from Izon Science Ltd., New Zealand. For all TRPS experiments, the lower and upper fluid cells contained 75 μL of electrolyte buffer, while the upper fluid cell additionally contained 35 μL of sample. A detailed description of TRPS methodology and equipment can be obtained from previous studies [44, 46, 48]. For all TRPS experiments, thermoplastic polyurethane nanopores (TPU, Izon Science Ltd.) were used. Detailed information on the TPU pores is presented by Sowerby et al. [49]. Carboxylated PS particle standards (Bangs Laboratories, USA) with nominal diameters ranging from 100 to 500 nm were used to calibrate the TPU pores. Particle concentrations were provided in %(m/v) solids (1% solids in water for all the above standards), and the respective nominal concentrations in particles per milliliter were calculated from the mean diameter and the density of PS (1.05 g mL^−1^) [24, 36]. In contrast to the standard sample preparation procedure described in “General sample preparation,” PS and PE standards were dispersed in phosphate-buffered saline (PBS) and FeOx samples were dispersed in 0.1 M Tris–HCl buffer (pH = 7.5). These electrolyte solutions were added to increase the electrical conductivity for reliable TRPS measurements. Surfactants including Tween 20 (0.03–0.1% m/v) and NovaChem100 (0.1% m/v) were added to the Tris–HCl buffer to facilitate particle dispersion.

### Disk centrifugation or centrifugal liquid sedimentation (CLS)

CLS relies on measuring the sedimentation velocity of particles in a liquid medium using the line start strategy and resulting in the determination of the Stokes diameter of particles [50]. Particles are injected into the center of a spinning, transparent disk containing a density gradient liquid, and their settling time is determined by measuring changes in light transmission at a detector position close to the perimeter of the hollow disk. The sedimentation velocity of the particles depends on their size, shape, and density. Thus, determining size distributions from disk centrifugation sedimentation time measurements according to Stokes law needs prior knowledge of the particle density (chemical composition and in some cases crystal phase). The resulting size distribution is absorbance based and can be transformed to mass- and number-based distributions with the help of further additional input data on optical properties at the applied wavelength [51, 52]. Shape also affects sedimentation speed; therefore, the instrument manufacturer suggests correcting the apparent density of the particles following a semi-empirical equation to gain proper equivalent sphere diameter data [53].

Experiments were performed using a UH 24000 disk centrifuge (CPS Instruments Europe, Netherlands) equipped with a sedimentation-type disk and a 405-nm light source. A 0–8% sucrose gradient in water (9 steps, each 1.6 mL) was applied at 22,000 rpm rotational speed. Polyvinyl chloride NPLs (*d* = 237 nm) were used as a calibrant before each injection. For non-spherical particles (i.e., FeOx nanorods), the apparent density was further adjusted following the instructions of the instrument manufacturer, considering the known aspect ratio of the particles. An aliquot (~ 150 µL) of a diluted sample (FeOx100 20 × , PS1-3 and FeOx2000 10 ×) was injected into the disk. The amount of injected sample volumes was estimated gravimetrically [54].

### Field-flow fractionation (FFF)

FFF–MALS is a fractionation technique, based on different physical properties, including particle diffusivity, coupled with an online multi-angle light scattering detector (MALS). MALS is used to measure the intensity of the scattered light at different angles. The angular-dependent scattering intensities can be used to calculate the radius of gyration (*R*_g_) [55]. The hyphenation with FFF reduces the risk of an overestimation of large particles in polydisperse NPL samples, which is typical for many light scattering–based methods. Furthermore, FFF can be hyphenated with various detectors (e.g., RM, single-particle inductively coupled plasma mass spectrometry, NTA, DLS) [30, 56, 57] to measure a wide range of complementary particle physicochemical properties.

#### Centrifugal field-flow fractionation (CF3)

CF3 analysis was conducted across two different laboratories on different instrumental setups to check the robustness of the hyphenation of FFF–RM. In both cases, the carrier liquid was prepared by adding 0.0125% (v/v) NovaChem100 (PN, Germany) to ultrapure water, which was obtained from a Milli-Q system (Integral 5 system, Merck KGaA, Germany) and filtered with a vacuum filtration unit through a 0.1-µm pore membrane (Durapore, Merck Millipore Ltd., Ireland).

After preparation, all samples were fractionated by a CF3 system from Postnova Analytics (CF2000, PN, Germany). The system included an autosampler (PN5300) and was hyphenated to an UV–vis detector (PN3211) and a MALS detector (PN3621, 21 angles). The nominal channel height was 250 µm. The UV absorbance was recorded at a wavelength of 254 nm. The instrument control and data evaluation were performed by the CF2000 control software (Version 1.0.2.7, PN, Germany). The angular-dependent scattering data were evaluated using 20 active angles in the range of 12° to 164°. Spherical models were used to fit the scattering data and obtain the radius of gyration (*R*_g_).

##### Laboratory 1

The FeOx100 and FeOx2000 NP stock suspensions were first sonicated for 15 min to re-suspend the particles and homogenize the suspension. After being diluted × 10 in carrier liquid, the suspension was tip-sonicated for 1 min with a 2-mm probe (UP200St, Hielscher Ultrasonics GmbH, Germany). To avoid excessive heating, the pulse mode (0.5 s on and 0.5 s off) was used. The tip was cleaned with water and ethanol to avoid sample contamination. This resulted in the formation of a clear, orange-colored suspension of FeOx100 and a clear, red-colored suspension of FeOx2000. The stability of the suspension was monitored with batch-DLS measurements.

The MALS detector angles were normalized with respect to the 90° angle using a fractionated PS particle standard (Nanosphere™ 3125A, ThermoFisher Scientific, MA. USA) with a nominal diameter of 125 nm. In contrast to the 20 angles used for all other samples, 21 detector angles ranging from 7 to 164° were used for PE1. All samples were fractionated using the same fractionation method. A detector flow rate of 0.5 mL min^−1^ was applied. Sample relaxation was performed for 5 min at an initial rotational speed of 4900 rpm. After sample relaxation, the elution profile consisted of a 15-min-long constant elution step at 4900 rpm followed by a 90-min-long exponential decay down to 60 rpm. In a third elution step, 60 rpm were kept constant for another 30 min. Both FeOx samples were analyzed by an optimized CF3 method using a lower initial speed of 3000 rpm to reduce interactions with the accumulation wall (i.e., increase sample recovery) and taking advantage of the higher density of these samples compared to the NPL samples. After a constant rotational speed phase of 10 min, the rotor speed was decreased exponentially over 66 min to 61 rpm, followed by another 20 min at constant speed. A rinse step of 15 min was used to remove any potential larger agglomerates and to minimize carry-over effects. The recovery of CF3 measurements was determined according to ISO/TS 21362 [58] by calculating the ratio of the peak area after fractionation to the peak area of a direct injection in the absence of a separation field. PE1 (non-spherical agglomerates) and FeOx2000 (rods) data were analyzed using a random-coil model to account for the non-sphericality and the higher aspect ratio. In MALS data analysis, the angular scattered light can be described by a function that takes the angular scattering of different geometries into account. The mathematical description used in the random-coil model is able to best fit the strong forward scattering contribution that was observed for this kind of samples. In the case of PE1, larger agglomerates might have been present, which show a stronger scattering contribution in forward direction.

##### Laboratory 2

Dilutions of the stock suspensions were prepared according to the general procedure (“General sample preparation”) using the carrier liquid. For dispersion, an ultrasonic bath (SONOREX SUPER RK 514 Ultra sonic bath, BANDELIN electronic GmbH & Co. KG, Germany) was used. Two different tri-modal particle mixtures were analyzed by online CF3–RM (Mix RM1, containing different polymer particles, and Mix RM2, containing polymer and inorganic particles). A detailed description of the Raman setup used can be found in “Raman microspectroscopy and hyphenation with field-flow fractionation.”

For testing the online coupling of the CF3 to the Raman microscope, the channel was bypassed and optical trapping (OT) was first investigated without any separation. For all other measurements, the channel was not bypassed. In OT, the optical forces (scattering and gradient force) of a focused laser beam can trap particles in the micro- and nanometer range at a certain position [59]. This enables the analysis of NPLs in suspension where particles would otherwise diffuse or be flushed out of the focus of the laser before sufficiently intense signals can be acquired. In all experiments involving OT, the particles were pushed against the bottom of the flow channel (2D-OT) to allow for a more stable trapping [30]. Further information on the OT setup can be found in the SM. To enable the hyphenation, a custom-built aluminum flow cell was used. It consisted of a metal base (Figure S1) on which a flow channel (1.5 cm × 1.5 mm) confined by an in-house-manufactured PET spacer with a height of 350 µm and topped with a glass coverslip (thickness 170 µm, Carl Zeiss Microscopy GmbH, Germany) that was attached using double-sided tape (thickness 50 µm, 3 M, USA). The fractionation method used an injection time of 4.75 min, a relaxation time of 3 min, and a maximum rotational speed of 3500 rpm with an exponential decay profile over 95 min. The detector flow was set to 0.2 mL min^−1^. Agglomerated fractions of samples were evaluated using a random-coil fit model. For further information on the evaluation of MALS data, see Schwaferts et al. [30].

#### Asymmetrical flow field-flow fractionation (AF4)

##### Instrumental setup

AF4 was conducted using a AF2000 Multiflow FFF (PN) coupled with a MALS instrument (PN3621 MALS Detector) and a UV Absorbance Detection System (Shimadzu SPD-20A/20AV). The instrument included the necessary isocratic pump(s), degasser, autosampler injectors, and automatic fraction collector. The following conditions were used for the analyses: (i) sample injected volume, 50–100 µL of undiluted sample (total injected mass between 0.5 and 10 µg); (ii) membrane, 10 kDa regenerated cellulose; (iii) mobile phase, NovaChem100 0.0125%; (iv) spacer, 350 µm; (v) injection flow, 0.2 mL/min; and (vi) detector flow, 0.5 mL min^−1^. In standard AF4 mode, focusing was performed at 2 mL min^−1^ for 6 min and a linear crossflow decay from 0.3 to 0 mL min^−1^ for 60 min was applied during the fractionation. Sample recovery (*R*%) was calculated according to ISO/TS 21,362 [58] by integrating the area under the UV–vis peak for each sample eluted, with and without (i) the applied crossflow and (ii) the focusing step [60]. The results with crossflow and/or focusing were compared to the results obtained without crossflow in order to calculate the *R*%.

##### Fractionation for offline pyrolysis GC–MS analysis

*Mono-modal samples:* For generation of the mono-modal samples, defined masses of each of the three materials (PS2, PS3, PSL60) were injected and fractionated with AF4 separately. Defined volumes belonging to the time windows specified for PS2 (24–34 min), PS3 (33–46 min), and PSL60 (20–26 min) were collected. To ensure a sufficient amount of material was available for the subsequent pyGC–MS analyses, multiple injections were made, and the same size fractions obtained from each injection were pooled. From the pooled suspension, aliquots of 5 mL were shipped for pyGC–MS analysis. Details are included in the SM, Table S4.

*Tri-modal samples:* For the generation of the fractions from the tri-modal sample MS1, a mixture of the same three materials (PS2, PS3, PSL60) was prepared. The fractionation procedure used was the same as for mono-modal samples described above. Specific details are provided in the SM, Table S5.

### Electron microscopy (EM)

Electron microscopy (SEM and TEM) is a group of imaging techniques based on the interaction of an electron beam with the sample. Size, shape, and surface characteristics can be obtained using these methods [4]. However, representativeness of the acquired data might not be sufficient, especially for heterogeneous samples, due to the low number of particles that can be measured. Furthermore, sample preparation by solvent evaporation may lead to agglomeration which can be avoided using more advanced techniques like environmental SEM or cryo electron microscopy [10].

SEM measurements were performed using a Sigma 300 VP Field Emission SEM (FE-SEM) from Carl Zeiss AG, Germany, equipped with secondary electron and in-lens detectors. A 30-µm aperture was used for all measurements. A 2.5 µL subsample of each diluted sample was drop-casted on silicon wafers and air-dried at room temperature.

For TEM, a double spherical aberration corrected cold FEG JEOL ARM 200FC TEM (Jeol Ltd., Japan), operated at 200 kV, was used. The sample dispersions were ultrasonicated for 5–10 min before a droplet was transferred to a copper TEM grid, coated with holey amorphous carbon. The particle size distribution, expressed by reporting the Feret min diameter, was measured by ImageJ using the NanoDefine Particle Sizers Plugin.

### Pyrolysis gas chromatography mass spectrometry analysis (pyGC-MS) of AF4 fractions

PyGC–MS is a mass-based quantification method used to identify the chemical composition of organic samples. Plastics can be distinguished by their pyrolysis fragments using, e.g., a database of commercial polymers to identify them. Furthermore, pyGC–MS allows for mass-concentration analysis by calibration, although this needs to be performed for each material individually due to different pyrolytic efficiencies and the technique is unable to directly provide any indication of particle number [9, 14].

Analysis of the PS NPLs and PS NPL fractions from AF4 analysis with pyGC–MS was conducted by two independent laboratories to allow comparison of the method and a degree of validation of its reproducibility. Both laboratories employed Agilent GC–MS instruments fitted with identical Frontier Multi-shot EGA/PY-3030D microfurnace pyrolyzers that were operated in single shot mode. Slightly different furnace and interface temperatures were used at each laboratory according to the respective in-house methods. Full instrumental settings and methodological details for each laboratory are summarized in Table S6. A key challenge with pyGC–MS analysis of NPLs extracted from environmental samples is their reproducible transfer to the pyrolysis crucibles without significant loss of material. Here, a solvent extraction method was developed and assessed across the two laboratories using the PS fractions generated by FFF in “Asymmetrical flow field-flow fractionation (AF4).”

#### Laboratory 1

A calibration curve was prepared by dissolving solid PS spheres (Hawai’i Pacific University Polymer kit 1.0 (PS-HW)) in ethyl acetate overnight to reach the target masses in 20 µL. A volume of 20 µL of each calibrant solution was then transferred into the pyrolysis crucibles and left in the oven at 40 °C to completely evaporate the solvent. The calibration curve ranged from 0.5 to 10 µg PS (Figure S2). The peak area of the marker compound 2,4-diphenyl-1-butene (styrene dimer; m/z 91) was used for quantification (Figure S3). Details about the preparation of the calibration solutions can be found in the SM (Table S7).

To determine the recovery of the PS NPLs after solvent extraction, stock suspensions of PS2, PS3, and PSL60 with the theoretical concentrations reported in Table 4 were diluted in 5 mL of 0.0125% NovaChem100 to reach a final theoretical absolute mass of 0.8 µg, 1.6 µg, and 1 µg in the pyrolysis crucibles for PS2, PS3, and PSL60, respectively. After evaporation of the liquid phase (overnight on heating blocks at 70 °C) and reconstitution of the polymer film in 600 µL of ethyl acetate under vigorous vortexing and bath sonication (two cycles of 2 min and 10 min each, respectively), an aliquot of 60 µL (1/10 of the total volume) was then transferred to the pyrolysis crucible. Three replicates were prepared for each material, and the samples were analyzed after complete evaporation of the solvent. The recovery of PS NPLs after the sample processing step was determined by dividing the experimentally determined PS masses after the sample work-up procedure by the known amount spiked into 5 mL of 0.0125% Novachem100.

Once the method had been established and acceptable recovery values determined, the PS-containing AF4 fractions (“Fractionation for offline pyrolysis GC–MS analysis” and detailed in Table S4 and S5) were analyzed. Samples (5 mL) were brought to dryness by overnight evaporation on heating blocks at 70 °C. The deposited PS film was then reconstituted in 600 µL of ethyl acetate under vigorous vortex stirring and bath sonication (two cycles of 2 min and 10 min each, respectively). An aliquot of 60 µL (1/10 of the total volume) was subsequently transferred to the pyrolysis crucible, and the sample analyzed by pyGC–MS following complete evaporation of the solvent.

#### Laboratory 2

A calibration curve was prepared by dissolving PS reference material (PS PTX300.00 < 1000 µm, Carat GmbH, Germany) in dichloromethane (DCM) at a concentration of 1000 µg mL^−1^ and vortexing at room temperature. Dilutions were made in DCM (1–1000 µg mL^−1^) and 10–50 µL were spiked into pyrolysis crucibles to obtain calibration masses in the range 50 ng to 10 µg. Calibration standards were run before and after each sample set, and the average response of duplicate injections was used for quantification. The peak area of styrene (m/z 104) was used for quantification against an external calibration curve.

Samples of the PS3 stock suspension were diluted (50 µL to 50 mL 0.0125% NovaChem100) and spiked into 2.5 mL 0.0125% NovaChem100 or directly in pyrolysis crucibles (50 µL in each). Three replicate samples were prepared for each treatment. The recovery across the sample preparation step was calculated as the percentage compared to PS directly spiked into crucibles. Once the method had been established and acceptable recovery values determined, the PS-containing FFF fractions were analyzed. Samples (2.5 mL) were transferred to glass vials and evaporated to dryness at 90 °C. The residual PS film was reconstituted in DCM using sequential triplicate extractions (~ 0.5 mL each time). Each DCM extract was reduced to approximately 50 µL and transferred to a pyrolysis crucible, for further evaporation. The combined extracted samples were analyzed by pyGC–MS after complete evaporation of the solvent.

### Raman microspectroscopy (RM) and hyphenation with field-flow fractionation

Although RM is currently one of the most powerful techniques for microplastics analysis [9, 61, 62], single polymer particle analysis at the nanoscale is difficult/challenging due to the diffraction limit of visible lasers. As such, NPL samples must be analyzed in bulk, which leads to challenges for complex samples (e.g., the presence of multiple polymer types). Another approach is the detection of dielectric particles, like NPLs, in suspension enabled by OT [63]. When hyphenated with FFF–MALS, RM allows for size-resolved chemical analysis of NPLs and particles in the size range of 100 nm to 5 µm [30].

For the online coupling of FFF to RM, an *alpha300 apyron* confocal Raman microscope (WITec GmbH, Germany, equipped with a 532-nm DPSS laser) was used. The microscope was equipped with a water immersion objective from Carl Zeiss Microscopy GmbH, Germany (63 × , “W Plan-Apochromat” series, N.A. = 1.0). The spectrometer (UHTS600 for VIS, 600 mm focal length) attached to the Raman microscope was equipped with a grating with 300 lines mm^−1^. A CCD camera (DU970N-BVF, Andor Technology Ltd, Northern Ireland) was used as a detector. All online measurements were performed using the time series mode with 10 s spectrum integration over the whole time of particle injection and separation. While a spectrum was recorded in the range of 100–3785 cm^−1^, only the intensity of one suitable Raman band per material was evaluated (TiO_2_: 146 cm^−1^, PMMA: 812 cm^−1^, PS: 1000 cm^−1^, PE: 2890 cm^−1^). However, it was ensured that the materials can be correctly identified by the presence of further Raman bands. To ensure a stable focus over the whole measurement duration, the *TrueSurface MkIII* module (WITec GmbH, Germany) was also enabled for online-coupled separation measurements. The laser was switched off for 5 s every 55 s to preserve particle fractionation. The same setup was used for batch measurements, but the objective was exchanged for an EC Epiplan-Neofluar HD DIC (100 × , N.A. = 0.9) from Carl Zeiss Microscopy GmbH, Germany. The samples were dried on aluminum foil. The resulting spectra were baseline-corrected using a rolling ball algorithm (150 pixels).

## Results and discussion

### Batch methods for particle characterization and quantification

#### Particle size distribution by dynamic light scattering, nanoparticle tracking analysis, and tunable resistive pulse sensing

Batch DLS, as the most commonly used ensemble technique, as well as NTA and TRPS as single-particle approaches [45], were selected for the measurement of the particle size distribution, the average particle size, and polydispersity of the selected samples (Table 2). For samples comprising monodisperse spherical particles, such as PS1 and FeOx100, the mean size values measured by the three selected methods were in good agreement. However, significant discrepancies in the measured mean size values were detected when measuring moderately or highly polydisperse samples (e.g., PS3 and PE1) and samples comprising non-spherical particles (e.g., FeOx2000). Unsurprisingly, the sizes obtained by DLS for all three samples are significantly larger than the other techniques and reflect the particle-size-dependent light scattering intensity [64]. Furthermore, the variance in particle size measured between laboratories for DLS measurements is significantly higher than that for monodisperse samples (Table S8). DLS cannot be considered a suitable method for the analysis of polydisperse or non-spherical particles, especially when based on the cumulant analysis. In the current study, the lack of reproducibility between laboratories and discrepancies with the results obtained by other measurement techniques confirmed this [65, 66]. For PE1, a significant difference in the measured size values was also detected between NTA and TRPS. This is possibly due to the different media used for sample dispersion prior to measurement. In contrast to NTA and DLS, a conductive dispersion medium is required to guarantee sample conductivity for TRPS measurements. The salinity in the media may lead to particle agglomeration, even if a surfactant is used to facilitate particle dispersion. Agglomeration of PE1 during TRPS measurements was confirmed by the results with the particle size obtained by TEM measurements (Figure S4), which shows that PE1 is composed of spherical particles with a mean diameter of 145 nm (*d*_10_ = 68 nm, *d*_90_ = 205 nm), comparable to the size values measured by NTA.

**Table 2:** Average particle size expressed as hydrodynamic diameter (TRPS, NTA, DLS), Stokes diameter (CLS), or diameter of gyration (*D*_g_) and polydispersity expressed as (*d*_10_, *d*_90_) or PDI determined by orthogonal sizing methods. For the DLS and MALS results, an average of both laboratories and the variance between them are given. The expected diameter for each sample is reported in Table 1. No certified nominal values for particle size are available for the samples

Sample	*d*_TRPS_ (nm)		*d*_NTA_ (nm)		*d*_DLS_ (nm)		*d*_CLS_ (nm)		*D*_g,MALS_ (nm)^2^	
	Mean (SD)^1^	(*d*_10_, *d*_90_)	Mean (SD)^1^	(d_10_, d_90_)	*Z*-average (var [%])	(PdI)	Mean (SD)^1^	(*d*_10_, *d*_90_)	Mean (var [%])	(*D*_g,min_, *D*_g,max_)
PE1	568 (14)	(419, 780)	147 (7)	(83, 229)	842 (67.2)	(0.513)	n.d.^3^	n.d.^3^	772^4^ (5181)	(162, 966)
PS1	143 (0.6)	(118, 186)	148 (4)	(110, 194)	141 (0.1)	(0.009)	118 (4)	(101, 144)	108 (15.3)	(92, 210)
PS2	n.d.^6^	n.d.^6^	140(1)	(116, 153)	154 (12.8)	(0.010)	111 (2)	(107, 126)	112 (6.3)	(102, 158)
PS3	255 (3)	(198, 324)	256 (2)	(175, 328)	338 (0.2)	(0.143)	221 (5)	(169, 268)	236 (4.6)	(166, 444)
FeOx100	154 (6)	(91, 230)	162 (3)	(125, 208)	173 (1.1)	(0.106)	165 (4)	(133, 192)	154 ^5^	(132, 312)
FeOx2000	139 (4)	(105, 170)	185 (7)	(137, 244)	254 (12.5)	(0.074)	161 (2)	(128, 192)	186^5^	(152, 432)

*n.d.* not determined

^1^Calculated from 3 repeat measurements

^2^The range was determined by evaluating the radius of gyration (*R*_g_) at 10% intensity of the MALS 90° signal

^3^Not possible to measure in standard setup due to the particles floating

^4^This *D*_g_ value was determined with different fit models by each laboratory

^5^The FeOx samples were analyzed by laboratory 1 only

^6^Not determined due to the limited amount of sample available

EM methods are the only approaches that are able to distinguish larger particles from agglomerates and that provide information on particle morphology, which is especially useful for rod-shaped particles like FeOx2000 and for irregularly shaped plastic particles that are commonly found in environmental samples. However, sample preparation using solvent evaporation might lead to the formation of agglomerates, which cannot be distinguished from previously existing agglomerates. Furthermore, the Eu-doping of PS2 can be imaged due to the high resolution of TEM. SEM and TEM images of selected samples can be found in the SM (Figure S4 and S5).

#### Particle size distribution by centrifugal liquid sedimentation

**Fig. 1:**
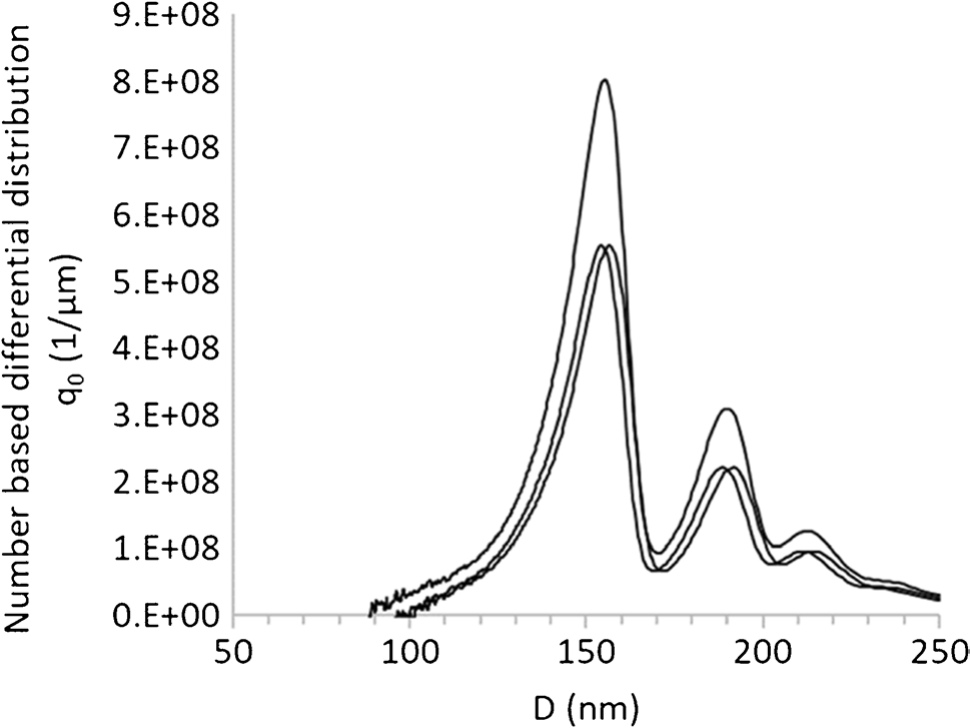
Number-based size distributions (3 replicate measurements) of spherical iron oxide particles (FeOx100) obtained by CLS analysis revealed the presence of particle multimers

For PS samples, CLS consistently measured a smaller mean size than the batch techniques (Table 2), which is possibly due to the high resolution of the method compared to batch techniques. Analysis of the FeOx100 particles by CLS provides additional insight into particle agglomeration, revealing three distinct size fractions representing different multimers (Fig. 1). PE1 could not be analyzed with the CLS setup due to the particle density being lower than that of the medium. A technical solution developed for these kinds of samples (“low density” disk) exists [67] and protocols are available, but the analysis of mixtures of particle populations with densities higher and lower than that of the liquid gradient cannot be performed contemporarily. Overall, CLS generally offers fast, well-reproducible, excellent size resolution [24, 54, 68] for measuring the size distribution of particles where former knowledge on the above-mentioned parameters is available. However, the technique is limited in applicability to environmental samples which will typically contain a mixture of unknown polymer types.

#### Particle concentration

Particle concentration was determined using TRPS, NTA, and CLS. Both TRPS and NTA count particles individually, generating number-based particle size distributions directly. As CLS concentration measurements are based on the determination of optical extinction, the technique produces light intensity–based particle distributions. To convert this into volume- and then number-based particle distributions (finally number concentrations), the material-specific refractive index and absorption values at the wavelength of the light source need to be known. The extinction efficiency of the particles is a sum of their absorption and scattering efficiency, the first scaling with particle size to the 3^rd^ power, and the second scaling with particle size to the 6^th^ power. The complex shaped, size-dependent extinction efficiency function is calculated by applying the Mie theory in the instrument software, which also allows the introduction of a shape factor in order to consider shape-related scattering properties. As an example, Figure S6 shows a comparison between the applied extinction efficiency (Qnet) functions for spherical PS and FeOx particles.

The current study found the concentration determined by different methods to be in fair agreement for most samples (Table 3 and Figure S7). The only exception was the very polydisperse PE1 sample, where the size range over which the polydisperse particle concentration is measured differs between methods. An overview of the dilution factors, including the measured concentrations, is given in Table S9. However, each of the techniques used in the study have their own set of limitations. For example, NTA camera settings do not allow for a broad range of particle sizes and refractive indices, which affects the measurable size range [69]. A possible solution to this problem is the coupling of NTA to separation techniques like FFF, which can produce narrower or defined size ranges [56]. For TRPS, various pores with different sizes might have to be used to cover the full size range of a polydisperse sample, adding more complexity to the measurement. For CLS, small particle sizes and the density of the particles compared to the gradient are limiting factors. For samples with very small particles (e.g., < 20–40 nm [36]), some of these techniques may not adequately cover the lower end of the size distribution, leading to diverging results. For example, it has been reported that NTA can overestimate particle concentrations compared to other methods [36, 70, 71], although such a trend was not obvious in this study. Interestingly, the agreement across all methods was significant for the concentration of the non-spherical sample FeOx2000, suggesting that three methods are comparable for quantifying particles independently of their shape.

**Table 3:** Average particle concentration and the percentage coefficient of variation (CV%) of the stock solutions calculated over > 3 replicates determined by TRPS, NTA, and CLS. The measured particle density for each sample is reported in Table 1. No certified nominal values for particle concentration in particles per milliliter are available for the samples. The expected concentrations, reported as particle mass value, are summarized in Table 1

Sample	*C*_TRPS_		*C*_NTA_		*C*_CLS_	
	Mean [mL^-1^]	(CV %)	Mean [mL^-1^]	(CV %)	Mean [mL^-1^]	(CV %)
PE1	3.33*10^10^	(7.5)	4.34*10^12^	(3.7)	–^1^	–^1^
PS1	1.60*10^13^	(10.1)	4.08*10^13^	(8.6)	4.17*10^13^	(25)
PS2	–^2^	–^2^	3.80 *10^13^	(0.6)	4.26*10^13^	(1.5)
PS3	9.77*10^12^	(9.5)	1.97*10^13^	(17.9)	2.35*10^12^	(7.4)
FeOx100	1.80*10^10^	(9.6)	1.92*10^10^	(12.6)	3.58*10^9^	(3.5)
FeOx2000	4.78*10^9^	(9.6)	9.28*10^9^	(21.5)	4.66*10^9^	(7.1)

^1^Not possible to measure in standard setup due to the particles floating

^2^Not measured due to the limited amount of sample available

#### Chemical analysis by Raman microspectroscopy in batch mode

Raman analysis in batch mode provides a pre-screening of the chemical nature of the particles, as well as being able to identify their crystal structure and any particle doping. For example, batch RM analysis of PS2 was used to detect the Eu-doping as two broad signals at around 1850 cm^−1^ and 2500 cm^−1^ [72]. Furthermore, different polymorphs (hematite, α-Fe_2_O_3_; magnetite, Fe_3_O_4_) were identified in the FeOx samples. More detailed information on the chemical characterization of the samples can be found in the SM (Figure S8).

#### Mass-based concentration by batch pyGC–MS

In addition to the particle number concentration, the mass concentrations of PS2, PS3, and PSL60 were determined by batch pyGC–MS. Visual assessment of the different stock suspensions already indicated differences in particle concentration at the same level of dilution, which was confirmed by pyGC–MS analysis (Table 4). While the theoretical mass concentrations of PS3 and PSL60 were very similar to the experimentally determined values, a big discrepancy was observed for PS2, which was about 10 times lower. Sedimented particles in the stock suspensions of PS2 were difficult to re-suspend and maintain in suspension, which may explain the large difference between theoretical and experimentally determined concentrations.

**Table 4:** Comparison of the theoretical (synthesis yield) and pyGC–MS-determined concentrations of polystyrene in stock suspensions

Sample material	Concentration of stock suspension (theoretical) [µg µL^−1^]	Concentration of stock suspension (experimental) [µg µL^−1^]
PS2	160	12.8 ± 1.3 (*n* = 3)
PS3	160	208 ± 1.7 (*n* = 3)
PSL60	10	11.3

Overall, the particle number–based techniques (NTA, TRPS, and DLS) all appear highly suitable for determining particle number concentrations, with each technique exhibiting some degree of limitation. However, the main limitation common to all 3 techniques is their inability to distinguish polymer particles from other types of particles. This means the techniques have very limited application for determination of NPL concentrations present in environmental samples. PyGC–MS overcomes this issue by being able to determine the mass-based concentration of different polymers in a sample but is itself limited by being unable to provide any indication of how many particles and what size of particles the determined mass represents.

### Coupled techniques for particle characterization and quantification

A fractionation step, separating the monodisperse fractions of polydisperse samples by size and/or density combined to an online measurement of their size or chemical composition can greatly enhance the resolution and sensitivity of the measurement results. For this reason, in this work, CF3 and AF4 fractionation approaches have been tested in combination with online sizing analysis by MALS and/or by online chemical analysis by RM.

#### CF3–MALS: coupled analysis of fractionation and particle size measurements

CF3–MALS was selected to measure the particle size distribution after fractionation of polydisperse samples into narrow size distributions by separating the particles according to their size and density. Broad size ranges and high polydispersity values were observed for the PE1 and PS3 samples (Table 2). The size ranges for PS3 determined across the two laboratories differ only at the upper end, which might be explained by the different separation profiles used. Furthermore, different *R*_g_ values were determined for PE1, which is mainly attributed to a degree of agglomeration of the PE1 sample received by laboratory 1. For this material, a better fit of the laboratory 1 MALS data was observed using a random-coil fit, while for laboratory 2, a spherical fit was better. In general, spherical and random-coil fits result in similar *R*_g_ values. However, larger particles show stronger scattering contributions in a forward direction that can be better described with the random-coil fit.

**Fig. 2:**
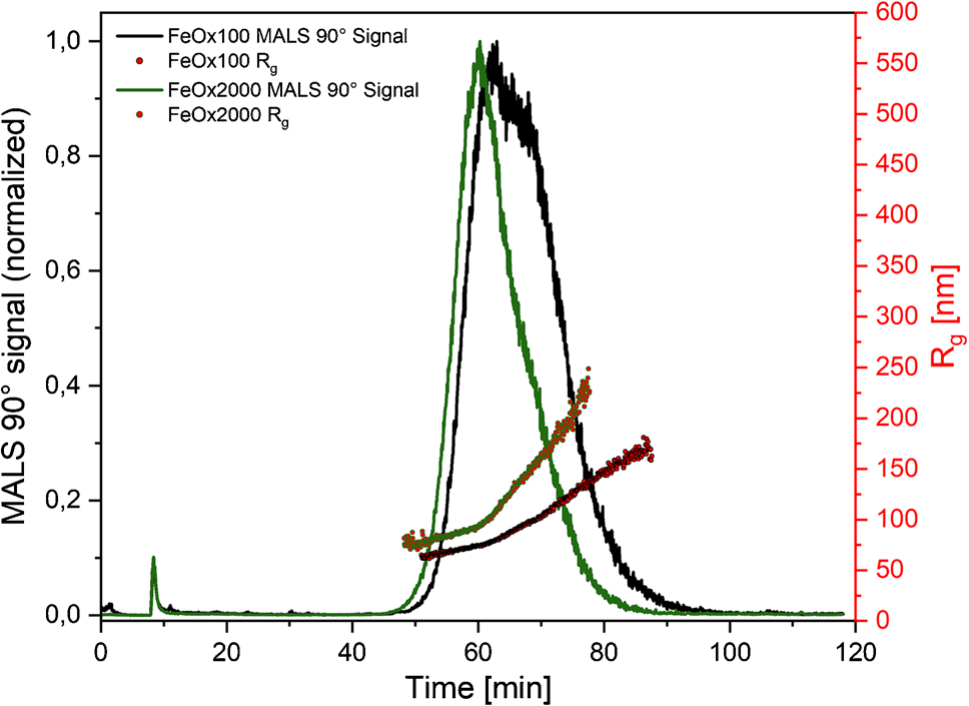
CF3**–**MALS data for both FeOx samples. For FeOx100, a broader distribution was determined by MALS, which agrees with the size distribution determination by CLS in Fig. 1

For the monodispersed PS1 and PS2 samples, a narrow size distribution was observed, with a small degree of agglomeration for PS2. The laboratory 2 data for these two materials showed visible agglomeration, which explains the relatively high full width at half maximum (FWHM) and the comparatively large size range. The spherical morphology of the PS samples was also confirmed by MALS evaluation due to the accordance of the scattering data with a spherical fit model. Furthermore, high repeatability between replicates was indicated by small variations (< 2.8%) in retention times. This is supported by low standard deviations for additional criteria, such as FWHM, *R*_g_ at peak maxima, and recoveries (Tables S10 and S11). For the FeOx100 sample, the spherical shape of particles was clearly confirmed by the agreement of the scattering data with the spherical fit model, in line with morphology detected by the TEM images. The *R*_g_ size distribution ranged from around 66 nm up to 156 nm, with 77.4 nm ± 1.1 nm at the peak maximum. MALS and UV–vis data further suggested a broader distribution, as shown by the fractograms in Fig. 2. The broader size distribution of FeOx100 was also observed with CLS, which offers even higher resolution. Additionally, the rod-like particles of the FeOx2000 sample also yielded a broad size distribution, ranging from around 76 nm up to 216 nm, with the *R*_g_ at the peak maximum being 92.8 nm ± 0.6 nm. Furthermore, high recoveries from around 84% up to 101% for all PS samples, FeOx samples, and the PE1 sample were obtained (Table S10), suggesting the CF3 method was able to characterize the complete size distribution of all samples. However, the technique might have limitations with a complex mixture of NPLs extracted from an environmental sample owing to the lack of information on particle composition.

#### CF3–RM: online-coupled fractionation and chemical analysis

In addition to the physical measurements provided by CF3–MALS, CF3–RM was tested as a complementary online-coupling approach for the chemical identification of particles in complex mixtures. FeOx2000 could not be detected by online RM with any of the tested parameters (flow rate: 0.1–0.2 mL min^−1^, laser power: 40–50 mW), most likely due to the lower trapping efficiency for non-spherical particles [73–78], and so this material was excluded from further experiments. It was also found that agglomerates are more difficult to detect. For the remaining particle types, including PE1 and PS2 (as representatives of the NPL samples), PMMA, and TiO_2_, a good balance between particle separation and detection/identification was achieved at a flow rate of 0.2 mL min^−1^ and a laser power of 50 mW, especially as PE1 proved difficult to detect/trap at lower laser powers. This is probably due to its lower density compared to other polymers, which results in a lower moment of inertia that could lead to PE being pushed out of the trap more easily by the hydrodynamic force [79].

**Fig. 3:**
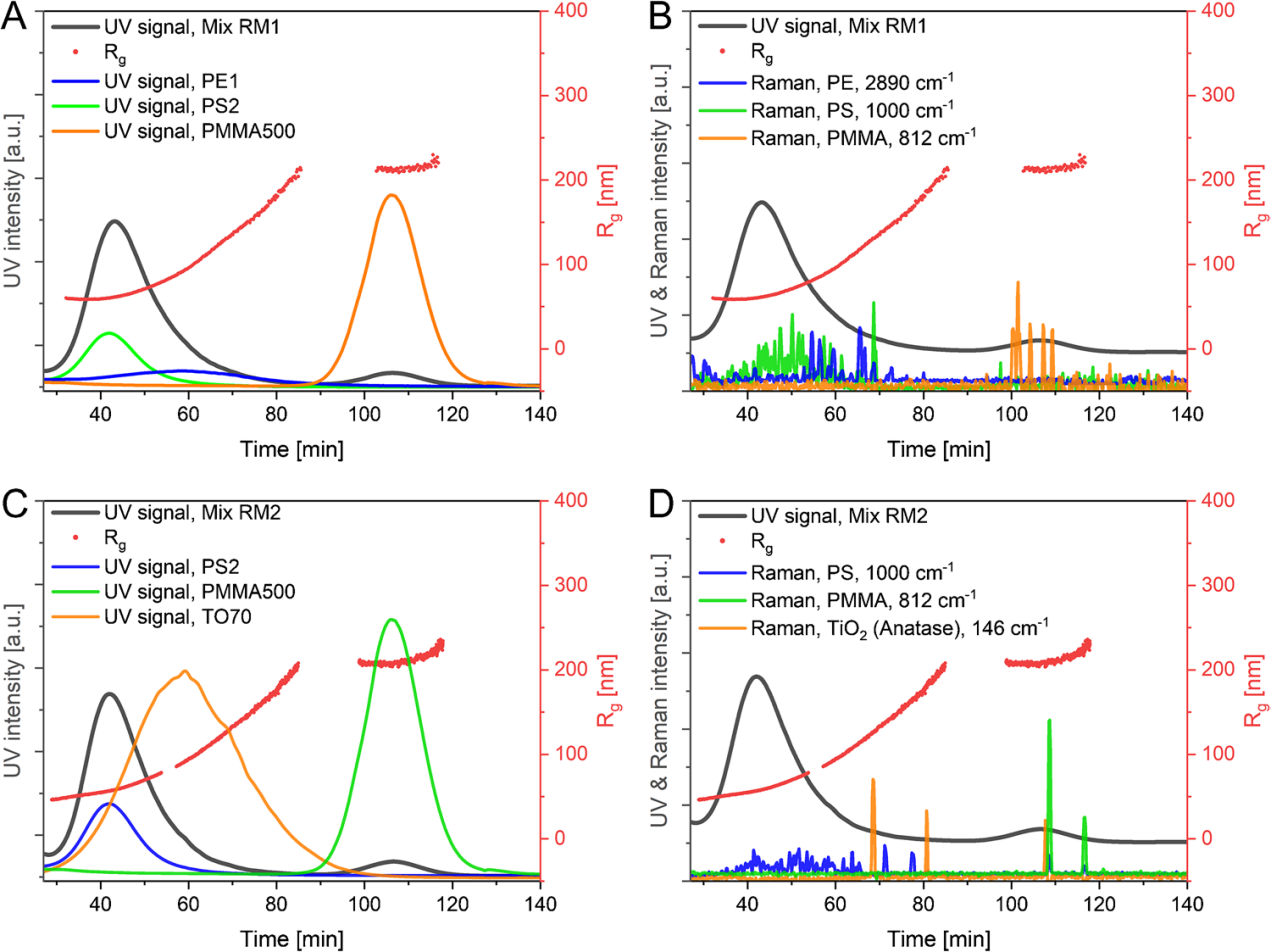
Online CF3**–**RM analysis of different particle mixtures. Separation of mixture RM1 (top) and mixture RM2 (bottom). The time of all coupled detectors was corrected to match the CF3 retention time. The radius of gyration (*R*_g_) determined by MALS data using a spherical fit is shown as red dots. **A** and **C** show the UV–vis data for the two mixtures including the UV–vis data for the single components, while **B** and **D** show the Raman data for the two mixtures. To improve clarity, only the intensity of one Raman band per material over time is shown

To test the capability of CF3–RM to distinguish particles with different chemical natures in a complex sample, two mixes of PE, PS, PMMA, and/or TiO_2_ were analyzed as reported in Table 1. When comparing the UV–vis signals of all the single components, the retention times were found to remain the same when present in both mixtures (vs the analyzed monodispersed samples of the same nature), indicating the particles do not strongly interact with each other. In the mixture RM1, only two peaks are distinguishable due to the retention time overlap of the polydisperse PE1 and the PS2 (Fig. 3A), while the PMMA500 eluted away from both PE1 and PS2. However, the MALS data indicated that the *R*_g_ values match well with those determined in the single component measurements. The Raman data (Fig. 3B) confirmed that the first peak at 43 min corresponds mainly to the PS2 sample, but that PE1 eluted over a broad time range. Surprisingly, it was not possible to detect PE when the majority of PS particles were observed. This might be either caused by the more difficult trapping of PE1 compared to PS2, or by a poor separation due to the high particle concentrations of PE1 and PS2. Importantly, these measurements show that also highly polydisperse NPL samples can be separated and analyzed using online CF3–RM coupling.

The mixture RM2 (PS2, TO70, and PMMA500) is especially suitable for Raman analysis as different TiO_2_ polymorphs (i.e., anatase and rutile) can be distinguished from each other. Again, only two peaks are distinguishable by UV–vis for this mixture, with the PS2 and TO70 overlapping (Fig. 3C). The polydispersity (possibly also agglomeration) of the TO70 material caused an increase in the determined *R*_g_ between the two UV–vis peaks. Raman analysis shows that particles in this mixture are less likely to be trapped, as fewer Raman signals, especially for anatase and PMMA, are obtained (Fig. 3D). This might be caused by heteroagglomeration of TO70 and the two NPLs, as agglomerates are more difficult to trap due to their non-spherical shape. Nevertheless, the results clearly illustrate that the simultaneous size fractionation and identification of polydisperse inorganic particles and NPLs is also possible using this technique. This indicates that CF3–RM coupling might be used for the analysis of NPLs in environmental samples without prior removal of all inorganic particles. However, particle–particle interactions (heteroagglomeration) must be considered in these mixtures. In summary, the online coupling of CF3 and RM can provide simultaneous size-resolved chemical information for NPLs and other particles in the size range of 100 nm–5 µm.

#### Offline-coupled AF4–pyGC–MS for particle characterization and quantification

**Fig. 4:**
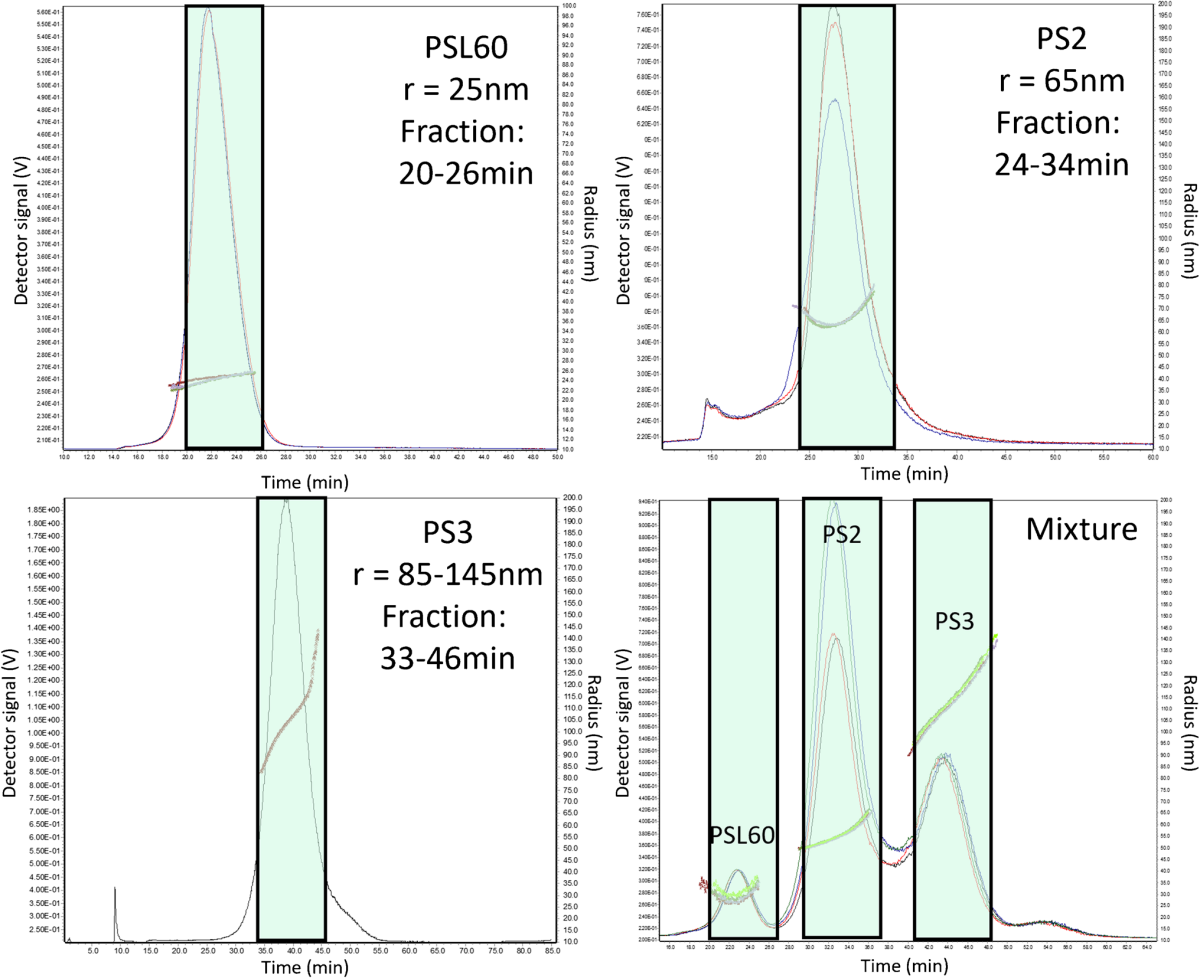
Sizing of the fractionated samples with multi-angle light scattering (MALS) and the time windows (green boxes) in which the fractions were collected. The time is shown as FFF retention time. The top left, top right, and bottom left figures show the fractionation of the individual samples (PSL60, PS2, PS3) including the radii of gyration (*R*_g_) while the bottom right figure shows the fractionation of a mixture of all three NPLs. The measurements were performed in triplicates (black, blue, red)

FFF followed by offline analysis of polymeric particles is an alternative to CF3–RM for chemical identification, also offering the possibility to semi-quantify the mass concentration of the particles belonging to different populations in the sample. For this reason, AF4–pyGC–MS was applied to the different PS samples and to their mixtures. In addition to the PS samples produced by SINTEF Industry, a well-known monodisperse NIST traceable standard, the PLS60, was included in the analysis. The AF4–MALS data from the fractionation shows mean *R*_g_ values of 25 nm for PSL60, 65 nm for PS2, and 85–145 nm for PS3, respectively (Fig. 4). This is in accordance with the data acquired from CF3 separation and shows good comparability between the two separation methods. Furthermore, all three different PS NPLs could be separated and isolated as individual fractions by AF4, with a minor overlap in retention times.

Recovery experiments to assess PS NPL losses during the sample work-up procedure were conducted by both participating laboratories. Laboratory 1 determined recoveries of 66 ± 8%, 75 ± 2%, and 69 ± 15% for PSL60, PS2, and PS3, respectively, based on triplicate analyses. Laboratory 2 determined losses during the sample work-up procedure for material PS3 only and observed a recovery of 80 ± 8% (*n* = 3). Considering the challenging concentration and sample transfer steps, the recoveries were considered satisfactory.

### Mono-modal samples

For PS2 and PS3, there was good agreement between the two laboratories for the absolute mass of PS determined by pyGC–MS for each of the fractionated mono-modal samples produced by AF4 (Table 5). However, a ca. × 10 difference was observed for PS2, possibly reflecting the difficulties in re-suspending this material. The overall recoveries in relation to the re-determined concentrations were 33.8%, 43.2%, and 46.4% (laboratory 1) and 314.1%, 39.4%, and 31.7% (laboratory 2) for PS2, PS3, and PSL60, respectively. These values account for the determined losses during the sample work-up procedure and during the fractionation with AF4, as well as the incomplete collection of each fraction. With the exception of the recovery for PS2 in laboratory 2, all other values range between 32 and 46%, indicating that  > 50% of the NPLs were not recovered. This difference is not explained by the losses in the extraction and sample processing steps determined in the recovery experiments, suggesting other losses are occurring. One contributing factor may be the incomplete elution of the sample materials during the AF4 fractionation process, which may derive from particles eluting outside of the defined time windows. Comparison of the areas (UV signal) of the injected samples with and without crossflow matched almost perfectly. However, this approach assumes that all of the injected material is eluted when not applying any crossflow, suggesting that no deposition on the tubing and membranes is occurring. Another factor that may have contributed to the uncertainty in the determined recovery values is the selection of the calibration range. The highest point in the calibration curve was 10 µg of PS, which, considering an injection split ratio of 1:20 and using the styrene dimer as the marker compound, corresponds to the upper limit of response linearity above which signal saturation occurs. A modest saturation effect can be observed in the calibration curve depicted in Figure S2. This results in an overestimation of the determined PS masses and also of the recovery rates, which in this case may have to be diminished further by approximately 5%.

**Table 5:** Absolute masses and recoveries of polystyrene in mono-modal and tri-modal pooled fractionated samples determined with pyGC–MS (offline analysis)

	Laboratory 1					Laboratory 2					
Sample	Experimentally Determined *mass* [µg]	Sample work-up recovery [%]	Mass corrected by work-up recovery [µg]	Expected mass considering fraction recovery^1^ [µg]	Overall recovery [%]	Experimentally determined mass [µg]	Sample work-up recovery [%]	Mass corrected by work-up recovery [µg]	Expected mass considering fraction recovery^1^ [µg]	Overall recovery [%]	Ratio overall recoveries (lab2/lab1)
Mono-modal samples											
PS2	0.12	75	0.16	0.47	33.8	1.2	80	1.49	0.47	314.1	9.29
PS3	4.10	69	5.94	13.76	43.2	4.3	80	5.43	13.76	39.4	0.91
PSL60	4.90	66	7.42	16.01	46.4	4.1	80	5.08	16.01	31.7	0.68
Tri-modal sample											
Fraction PS2	0.86	75	1.15	0.91^1^	126.4	1.52	80	1.90	0.91^1^	208.8	1.65
Fraction PS3	0.60	69	0.87	5.20^1^	16.7	1.19	80	1.49	5.20^1^	28.7	1.72
Fraction PSL60	0.90	66	1.36	2.83^1^	48.1	1.78	80	2.23	2.83^1^	78.8	1.64
Sum of fractions	2.36 (sum)		3.38 (sum)	8.94 (sum)	37.8	4.49 (sum)		5.62 (sum)	8.94 (sum)	62.9	1.66

^1^Assuming fraction recovery of 90%

### Tri-modal samples

Overall recoveries in relation to the expected concentrations were 16.7%, 126.4%, and 48.1% (laboratory 1) and 28.7%, 208.8%, and 78.8% (laboratory 2) for PS2, PS3, and PSL60, respectively (Table 5). Both laboratories overestimated the total absolute mass of PS3 (+ 26.4%/ + 108.8%), while underestimating the mass of PS2 (− 83.3%/ − 71.3%). For material PSL60, laboratory 2 reached a good recovery of around 80%. An underestimation of the mass for each fraction was expected based on the results of the mono-modal sample analysis, but the results from the tri-modal analysis indicate that there are further complications arising when the particles are present in a mixture. As the extraction procedure is indiscriminate toward the different particles, the data suggest that the mixtures impact the separation capacity of the AF4 approach. This hypothesis is supported by the appearance of a minor fourth peak in the fractogram (Fig. 4).

PyGC–MS has become increasingly popular for the identification and quantification of polymers in various matrices, especially those in size ranges that are not amenable to spectroscopy-based techniques such as µFTIR and µRaman. In this study, it proved highly suitable for the identification of PS NPLs in fractionated samples, but only semi-quantification by mass could be achieved due to the variability in recovery (especially in a mixture). Furthermore, no direct information on particle size and shape can be extracted from pyGC–MS analysis. Other limitations of this technique include the relatively high limit of quantification and the need for specific solvents to extract NPLs, both of which differ for each polymer type. While the concentration of samples was achieved by solvent extraction and evaporation in the current study, an alternative approach could be to concentrate particles from the collected fractions on filters with small pore sizes (e.g., Anodisc™). If small enough, the filters can quantitatively be transferred to the pyrolysis crucible, although such an approach would be limited to particles that are quantitatively trapped on filters, potentially excluding analysis of smaller nanoparticles. Furthermore, the size of the filters is limited to the size of the pyrolysis crucibles, and they must be flexible to allow rolling or folding.

The analytical combination of FFF and pyGC–MS seems a promising approach for the identification and semi-quantitation of polymers in heterogeneous samples, but there is a need for further method development and optimization that specifically focuses on improved and reproducible fractionation, extraction, and quantification. Attention to addressing the source of the discrepancies between expected concentrations of NPLs collected in the AF4 fractions and the determined concentrations by pyGC–MS is needed going forward. Likely focus areas include assessment of particle suspension stability within the AF4 system and the potential for loss of particles adhering to collection vials and during sample transfer for analysis. While the current study utilized only PS NPLs, the analytical approach has the potential to be extended to a range of other polymers, depending on the identification of appropriate solvents to dissolve polymers for extraction and prepare calibration curves. Furthermore, the use of solvent combinations to allow simultaneous extraction of multiple NPL polymer types would need to be investigated.

### Comparison of different methods

Each technique evaluated in the current study has its own strengths and limitations in terms of parameters measured, ease of use, complementarity of the measured attributes, instrumental cost, range of applicability, and capability to measure polydisperse samples (Table 6). Monodisperse samples comprising pristine spherical NPL particles can be robustly characterized when combining a technique for measuring the physical properties (e.g., size, shape, and polydispersity by DLS, TRPS, NTA) and a complementary approach for chemical identification (e.g., RM or pyGC**–**MS). Many of the techniques are able to offer either number- or mass-based quantification, but each lacks the ability to do this comprehensively for NPLs. For example, number-based instruments are not able to distinguish between particles with different chemical compositions (e.g., polymer vs non-polymer) from each other. In contrast, mass-based techniques can only provide the total mass of a polymer or polymers in a sample, without being able to inform about the particle size or number. Therefore, a combination of at least two methods appears necessary to quantify the number of particles of a specific size and polymer type present in a sample. The situation is significantly more complicated when attempting to work with complex environmental samples that likely contain far more non-polymer particles than polymer particles. Effective separation and isolation techniques appear to be currently lacking (and may not be developed), meaning most samples will still contain a combination of polymer and non-polymer (matrix) particles at the point of analysis.

**Table 6:** Comparison of the complementary techniques used in this study. The measurands associated with each technique (particle size, morphology, particle concentration, and chemical identification) are described in the first 4 columns. In the technique-related properties column, applicability and limitations in terms of size range, shape assumption, performance in resolution cost per sample, and measurement complexity are classified. Measurement complexity is described (i) in terms of the technical expertise to develop a measurement protocol and (ii) according to the time per sample needed for routine measurements. Possible bias sources identified in this work and the complementary information obtained are summarized in the last two columns. Parameters such as the applicable size range, resolution, and performances in concentration measurements may vary when samples of different nature (e.g., inorganic samples) are tested. Qualitative parameter for quantification: low < moderate < medium < high

Techniques	Measurands				Technique-related properties [24]	Possible bias source	Unmask bias? Combined information?
	Particle size/polydispersity	Morphology	Concentration	Chemical identification			
DLS	Yes	No	No (only MA-DLS)	No	Size range: 1–800 nm Resolution: low Spherical shape assumed Cost per sample: low Technical expertise: low Time per sample: low (10 min/sample)	Overestimation of larger particles Low reproducibility for not spherical or polydisperse samples	*Unmask bias by combining orthogonal methods based on different physical principles:*
TRPS	Yes	No	Yes, particle/mL	No	Size range: 40 nm − 20 µm Resolution: very high Cost per sample: low Technical expertise: moderate Time per sample: low (15 min/sample)	Particle aggregation with high-salinity buffer used for the measurement experiences with PE	**Particle size:** DLS: only applicable to spherical monodisperse samples NTA, CLS, TRPS, and CF3–MALS allow a higher resolution when applicable
TEM, SEM	Yes	Yes	No	No	Size range: 1–800 nm Resolution: very high direct information on morphology Cost per sample: high Technical expertise: high Time per sample: high (3 h/sample)	Time consuming, counted number of particles is low compared with other techniques	TEM allows the direct determination of particle shape and distinguishing agglomerates of small constituent particles from those of larger isolated particles
NTA	Yes	No	Yes, particle/mL	No	Size range: 10–800 nm Resolution: high Spherical shape assumed Cost per sample: low Technical expertise: moderate Time per sample: low (15 min/sample)	Overestimation of larger particles	**Concentration:** NTA, TRPS and CLS provide comparable results for particle concentration measurements, when applicable **Chemical identification:** PyGC–MS allows for (semi-)quantification of NPLs
CF3–MALS	Yes	Indirect	(Yes, but dedicated concentration detectors, e.g., UV–vis are more convenient)	No	Size range: 10 nm–50 µm depending on the density of the analyte (MALS: 10 nm to approx. 1000 nm) Resolution: high Indirect information on morphology Cost per sample: moderate Technical expertise: moderate Time per sample: medium (2 h/sample)	Possible low particle recovery during fractionation, even if in this study particle recovery for PS, PE, and FeOx was satisfactory (> 70% in mass)	CF3–RM provides size-resolved information on NPLs and inorganic materials, with additional information (polymorphs, doping) in batch mode Density information from centrifugal methods (CLS, CF3) might contribute to identification
CLS	Yes	No	Yes, mass based	Indirect (density)	Size range: density dependent 10 nm–50 µm Resolution: very high Indirect information on density Density values needed to interpret the results Standard measurements not possible for density < 1.02 g/mL Cost per sample: moderate Technical expertise: moderate Time per sample: density and size dependent, low (10 min/sample)	Calibration is needed before each measurement; calibrant size and density affect results Particle density is needed for measuring particle size Measurement of low-density materials (such as PE) requires a specific setup due to its density Particle shape affects sedimentation speed Optical properties affect transformation of intensity-based data to mass-based data	
PyGC–MS	No	No	Yes, mass based	Yes	Size range: – Concentration range: 0.01–10 µg Cost per sample: low Technical expertise: high Time per sample: moderate (0.5–0.75 h/sample)^a^	Low recovery during sample preparation, no information on inorganic materials	
Batch RM	Yes	No	No	Yes	Size range: > 300 nm or bulk Concentration range: material and focal volume dependent Resolution: very low Cost per sample: low Technical expertise: low Time per sample: low (15 min/sample)	No quantification, no size information on NPLs	
CF3–RM	Yes	No	No	Yes	Size range: 100 nm–5 µm Concentration range: µg/L to g/L (sample dependent) Resolution: high Indirect information on morphology Cost per sample: moderate Technical expertise: high Time per sample: medium (2 h/sample)	No quantification, optical trapping limits the detection of small and non-spherical particles	

^a^The indicated duration of the (instrumental) sample analysis does not include the calibration procedure

Light scattering techniques such as DLS or NTA are most applicable to spherical particles, since they are based on an algorithm that assumes a spherical shape for the particles to calculate the particle size from the primary measurand, as evident from the variance between measurement results obtained for the medium diameter of the rod-like particles of the sample FeOx2000. As such, these techniques may not be suitable for characterizing and quantifying irregularly shaped NPLs, which are likely to be most common in environmental samples. The measurement of particle size and polydispersity of polydisperse samples is also challenging, especially for the commonly used light scattering techniques such as the batch DLS, as demonstrated by the lack of reproducibility experienced in the measurements of PE1.

Compared to the simple batch techniques evaluated in this study for measuring the particle size distribution, CLS offers better size resolution. However, CLS relies strongly on knowing the density (and shape) of the target particles to get reliable results, which cannot be known when attempting to analyze unknown NPL particles in an environmental sample. In this case, TRPS or NTA can provide a more independent size characterization of the total sample. Non-spherical particles are difficult to characterize with a single method, even if any assumption of the particle shape is made (TRPS, CLS). EM measurements are needed for direct measurement of particle morphology, but the technique is not suited to particle-by-particle characterization as the whole sample cannot be viewed at the same time. Limitations of the applicability of the different measurement techniques may also come from the specific chemical nature of the particles analyzed. For example, the analysis of PE particles by CLS requires a different (non-standard) disk due to the specific, low density of the material. The measurement of PE with TRPS was also found to be challenging due to the sample agglomeration induced by the saline media used for the measurement, an effect that is not detected when measuring PS or FeOx particles.

To increase the resolution and the sensitivity of the analysis, offline- or online-coupled (hyphenated) techniques can be used. As demonstrated in this work, the coupling of FFF to a light scattering detector (either DLS or MALS) helps to improve the intrinsic limitation of light scattering measurements in batch mode and also gives indirect information on particle shape. Interestingly, we demonstrated that CF3–MALS analysis on different instrumental setups showed high recovery rates and repeatability, even for particle types with very different chemical properties (e.g., PS, PE, and FeOx). The results indicate that CF3–MALS is a robust analytical approach for the measurement of the particle size distribution of polydisperse NPL particles of different compositions. Online coupling of CF3 with complementary detectors to analyze the chemical composition of the particles in addition to particle size appears to allow a combination of complementary physical and chemical information. This has the potential to be an extremely useful hybrid approach for analyzing complex mixtures of particles comprising different physical and chemical properties. As such, it has potential application in the analysis of NPLs in environmental matrices, but it should be noted that chemical characterization is not conducted on single particles, but rather the bulk material isolated in different FFF fractions.

Both pyGC–MS and RM can be used to identify the polymer type of NPLs. However, RM can also deliver information on the material of inorganic particles (or inorganic doping of NPLs) and distinguish different polymorphs. As online CF3–RM is dependent on OT, where the efficiency depends on particle size and shape, small or non-spherical particles are less likely to be detected compared to larger or spherical particles, showing some limitations in the sensitivity of the online coupling vs the batch mode approach. As a mass-based method, pyGC–MS can detect/identify and quantify NPLs independent of their shape and size, although the observed NPL losses associated with the fractionation process, sample extraction, treatment, and transfer mean semi-quantitation (instead of quantification) are a more accurate description at present. Such sample preparation issues, therefore, are still to be solved for AF4–pyGC–MS to ensure that the promising coupling of the two techniques can generate reliable data. Compared with the offline AF4–pyGC–MS analysis, online CF3–RM offers better time (and as a consequence size) resolution of the chemical analysis since there is no need for fraction collection.

### Environmental relevance and suitability

It is important to note that high concentrations (particle number/density) of NPLs and NPs were used in the development and validation of the different methods evaluated as part of the current study. Such an approach is common and typically necessary to ensure robust data is generated. However, the concentrations used in the studies may not reflect naturally occurring environmental concentrations of NPLs, although there is very little data currently available due to the lack of suitable techniques for their determination in environmental samples (especially complex matrices such as sediments and biota). The next step in the development of procedures for quantifying NPLs in environmental samples should involve the application of the most promising techniques from the current study to real samples. This should include the spiking of reference samples with relevant reference NPL materials at a range of concentrations to determine extraction efficiency and accuracy.

It is also important to highlight that the different methods have some specific limitations, as well as variations in the expertise required for method development and in the time required to conduct analyses. For example, the density of NPL particles will vary quite strongly depending on the specific polymer type each particle is comprised and can represent a limitation with some of the techniques described here. In particular, CLS is highly dependent upon knowing the density value of the polymer(s) present in a sample. As such, CLS can be a viable analysis and quantification method for samples containing known polymer types, but is unlikely to have strong potential for application to environmental samples containing an unknown mixture of polymer types at an unknown ratio to each other. Similarly, some of the techniques are quite high throughput (e.g., DLS and NTA) owing to the simplicity of the sample preparation (e.g., use of dispersions) and the rapid analysis times (seconds to minutes). In contrast, other techniques require much more advanced sample preparation and involve much longer analysis times and data processing times (e.g., TEM, pyGC–MS). In addition, the required level of detail regarding particle numbers within defined size classes can also have a strong impact on the amount of resources (time, instrumentation) required to generate the target data. For example, analysis and quantification of total NPLs present in a sample would not necessarily require the use of FFF-type fractionation into defined size bins and would therefore significantly reduce the cost and time required to analyze a sample (although at the expense of high-resolution data). An estimate of the time required to conduct each of the analyses is included in Table 6.

## Conclusion

For a broad physicochemical characterization and quantification of NPLs, combinations of different methods are not only needed, but also increasingly becoming available due to the method development in this field over the past few years. Coupled/hyphenated techniques offer complementary data that give improved insight into NPLs in more complex samples. In the case of online coupling, they can provide an extensive data set with only one set of measurements, as was demonstrated by the online coupling of FFF and RM to obtain size-related chemical characterization of NPLs. For specific in-depth information, such as high size resolution or advanced material characterization, offline techniques such as EM, CLS, TRPS and offline RM and pyGC–MS, are still needed. All of the individual techniques evaluated generally exhibited good agreement with each other, given that similar parameters were obtained. Only in the case of samples consisting of non-spherical and polydisperse NPLs were deviations observed, especially regarding their size.

However, all the techniques appear to have significant limitations with respect to the identification, characterization, and (especially) quantification of NPLs present in complex environmental samples. Unless sample preparation and pre-concentration techniques can be developed for a complete isolation of NPLs from other particulate materials present, none of the subsequent analysis techniques will be able to provide accurate data specifically for the NPLs, instead providing average values for all particles present (e.g., size, number). The coupling of multiple techniques, such as CF3–RM and AF4–pyGC–MS, appears to offer some advancement with the ability to separate specific size fractions for subsequent polymer identification, as well as mass-based semi-quantification in the case of pyGC–MS. The extension of the CF3–RM setup with an online concentration detector could allow for quantification in addition to the size and chemical characterization. However, further development of such methods, in conjunction with the sample preparation techniques, is needed to achieve a level of NPL identification and quantification comparable to that currently possible for plastic particles in the micrometer size range.

## Supplementary Information

Below is the link to the electronic supplementary material.

Supplementary file1 (PDF 625 KB)
