# Supplementary material for: Physicochemical characterization and quantification of nanoplastics: applicability, limitations and complementarity of batch and fractionation methods
Source: Anal Bioanal Chem. 2023 Apr 27;415(15):3007–31. doi: 10.1007/s00216-023-04689-5 (PMC10284950; doi:10.1007/s00216-023-04689-5)
Supplement: Supplementary file 1 — Supplementary file1 (PDF 625 KB) [file 216_2023_4689_MOESM1_ESM.pdf]

Supplementary Material

Physicochemical Characterization and Quantification of Nanoplastics: Applicability, Limitations and Complementarity of Batch and Fractionation Methods

M. J. Huber[1], A. M. Booth[2,*], I. Beer[1], I. Bianchi[3], R. Drexel[4], O. Geiss[3], D. Mehn[3], F. Meier[4], A. Molska[5], J. Parot[5], L. Sørensen[2], G. Vella[6], A. Prina-Mello[6], R. Vogel[7], F. Caputo[8,*], N. P. Ivleva[1,*]

*Corresponding authors:

Andy M. Booth (andy.booth@sintef.no)

Fanny Caputo (fanny.caputo@lne.fr)

Natalia P. Ivleva (natalia.ivleva@tum.de)

[1] Institute of Water Chemistry (IWC), Chair of Analytical Chemistry and Water Chemistry, School of Natural Sciences (NAT, Dep. Chemistry), Technical University of Munich (TUM), Lichtenbergstr. 4, D-85748, Garching, Germany

[2] Department of Climate and Environment, SINTEF Ocean AS, Trondheim, Norway

[3] European Commission, Joint Research Centre (JRC), Ispra, Italy

[4] Postnova Analytics GmbH, Landsberg am Lech, Germany

[5] Department of Biotechnology and Nanomedicine, SINTEF Industry, Trondheim, Norway

[6] Laboratory of Biological Characterization for Advanced Materials (LBCAM), Department of Clinical Medicine, Trinity Translational Medicine Institute, Trinity College Dublin, Dublin, Ireland

[7] School of Mathematics and Physics, The University of Queensland, QLD 4072, Australia

[8] Laboratoire National de Métrologie et d'Essais, France and (second affiliation) Department of Biotechnology and Nanomedicine, SINTEF Industry, Trondheim, Norway

Additional analysis of silica (SiO_2) samples

The silica samples (Table S1) were prepared according to the procedure for NPLs in 2.1.

Table S1: Overview over the silica samples analyzed by various batch methods for additional comparison. Sample description, including the supplier, the expected particle concentration, expected size, expected particle shape, measured particle density, chemical composition and list of the complementary techniques used in this work are reported for each sample.

Sample	Supplier	Expected Size (Shape)	Description	Techniques used for analysis
**SISN50**	nanoComposix, USA	50 nm (spherical)	Silica nanoparticles, monodisperse	TRPS, CLS, SEM, TEM, RM
**SISN100**	nanoComposix, USA	100 nm (spherical)	Silica nanoparticles, monodisperse	NTA, TRPS, CLS, SEM, TEM, RM
**SISN200**	nanoComposix, USA	200 nm (spherical)	Silica nanoparticles, monodisperse	NTA, TRPS, CLS, SEM, TEM, RM
**SISN500**	nanoComposix, USA	500 nm (spherical)	Silica nanoparticles, monodisperse	NTA, TRPS, CLS, SEM, TEM, RM
**SISN1000**	nanoComposix, USA	1000 nm (spherical)	Silica nanoparticles, monodisperse	TRPS, CLS, SEM, RM

The sizes and size ranges determined by TRPS, NTS, and CLS were in overall good agreement between the methods (Table S2). The greatest deviations were observed for SISN200 and SISN500. The addition of buffers for TRPS might lead to agglomeration, which results in larger sizes. For NTA, the camera settings used for the largest sample SISN500 might not have been optimal, which could result in mainly smaller particles being detected. In general, it is not possible to measure a broad size range with the same NTA setup. While the concentrations determined by these methods are in overall good agreement (Table S3), variances >50% were observed for SISN200 and SISN500. This can be explained by the different size ranges detected by TRPS, NTA, and CLS.

Table S2: Particle sizes (hydrodynamic diameter) and size distributions of silica samples determined by TRPS, NTA, and CLS.

Sample	d_{TRPS} Mean [nm] (d_{10},d_{90})	d_{NTA} Mean [nm] (d_{10},d_{90})	d_{CLS} Mean [nm] (d_{10},d_{90})
**SISN50**	59 (51,67)	-	54 (50,60)
**SISN100**	113 (99,129)	116 (92, 129)	102 (82,118)
**SISN200**	249 (199,321)	209 (164,229)	198 (158,221)
**SISN500**	527 (474,594)	407 (240,495)	485 (421,518)
**SISN1000**	1040 (958,1123)	-	989 (828,1061)

[1] Calculated from 3 repeat measurements.

Table S3: Particle concentrations of silica samples determined by TRPS, NTA, and CLS.

Sample	C_{TRPS} Mean [mL^{-1}] (CV %)	C_{NTA} Mean [mL^{-1}] (CV %)	C_{CLS} Mean [mL^{-1}] (CV %)	Variance between methods [%]
**SISN50**	$4.02*10^{12}$	-	$1.06*10^{13}$ (2.5)	20.3
**SISN100**	$1.43*10^{13}$ (2.2)	$8.07*10^{12}$ (1.8)	$2.84*10^{12}$ (5.0)	31.1
**SISN200**	$4.94*10^{11}$ (1.5)	$2.55*10^{12}$ (2.5)	$5.07*10^{11}$ (4.9)	66.6
**SISN500**	$5.18*10^{10}$ (3.8)	$2.56*10^{11}$ (26.7)	$3.78*10^{10}$ (6.4)	74.9
**SISN1000**	$1.01*10^{10}$ (8.1)	-	$5.69*10^{9}$ (8.6)	7.8

Supplementary material for the hyphenation of CF3 and RM

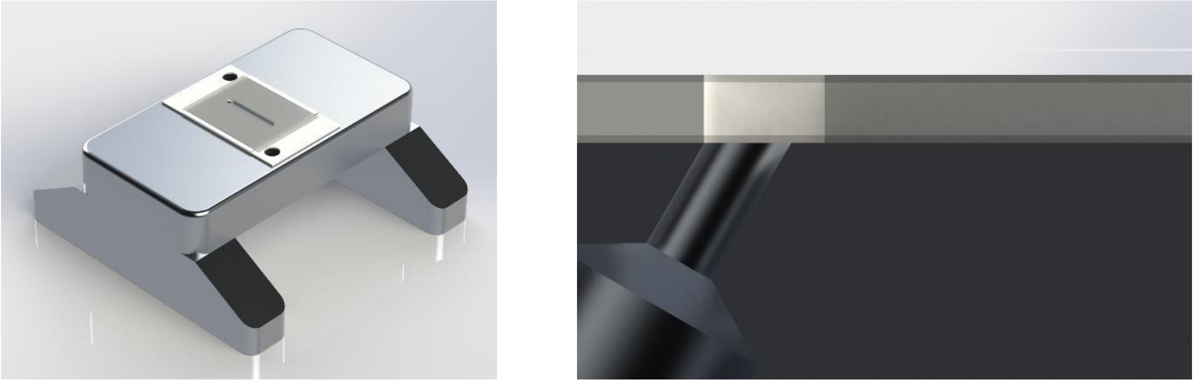

Figure S1: Schematic isometric and cross-section view (created using SOLIDWORKS 2021b) of the aluminum flow cell. In the cross-section view the dark grey layer at the bottom represents the aluminum body, while the alternating grey and light grey layers represent the double-sided adhesive tape and the PET spacer respectively. The cell is confined by a cover glass slide at the top. In all experiments the laser is focused directly above the aluminum surface.

To give an estimation of the ratio of measured and injected particles the ratio of cross section areas of the focal volume and the flow channel is calculated. The width and the height of the focal volume are assumed to be around 300 nm and 1 µm with an elliptic shape. Due to the optical forces, however, we assume the effective focal volume to be larger (600 nm and 2 µm). Thus, the resulting ratio of the channel cross section area and the focal area is $5.6*10^{-6}$. Assuming a trapping efficiency of 100% and a uniform particle distribution across the channel cross section, about 6 in a million particles are detected. This partly explains the high concentrations needed for reliable detection. Further improvements to the flow cell can improve this ratio.

Table S4: Procedure for the sample generation of mono-modal samples by AF4 fractionation for offline pyGC-MS analysis

	Sample Materials		
	PS2	PS3	PSL60
Concentration Stock-Suspension of PS NPLs experimentally determined by Py-GC/MS [mg mL^{-1}]	12.8	208	11.3
Dilution factor	1000	1000	100
Concentration Diluted suspension [µg mL^{-1}]	12.8	208	113
Volume injected in AF4 [µL]	50	100	100
Corresponding injected mass[µg]	0.64	20.8	11.3
Fraction recovery[1] [%]	74	86	85
Injected mass corrected by fraction recovery [µg]	0.47	17.89	9.61
Numbers of injections under the same conditions	3	3	4
Total mass in combined injections [µg]	1.42	53.67	38.42
Volume/fraction [mL][2]	5	6.5	3
Total volume of fractions [mL]	15	19.5	12
Concentration in pooled fractions [µg mL^{-1}]	0.095	2.752	3.202
Absolute theoretical mass in shipped 5 mL [µg]	0.48	13.76	16.01

[1] Percentage of eluted peak. Determined by dividing total peak area (UV signal) and area of peak area in elution window (Figure 4).
[2] Volume of collected fraction of single injection.

Table S5: Procedure for the sample generation of tri-modal samples by AF4 fractionation for pyGC-MS analysis

	Fraction		
	PS2	PS3	PSL60
**Concentration Stock-Suspension of PS NPLs experimentally determined by Py-GC/MS [mg mL^{-1}]**	12.8	208	11.3
**Dilution factor**	1000	1000	100
**Concentration Diluted suspension [µg mL^{-1}]**	12.8	208	113
**Volume added to mixture [mL]**	0.8	0.24	1.44
**Absolute mass in mixture [µg]**	10.24	49.92	45.2
**Concentration in mixture [µg mL^{-1}]**	7.11	34.67	31.39
**Volume injected in AF4 [µL]**		100	
**Correspondon Injected mass [µg]**	0.71	3.47	3.14
**Number of injections under the same conditions**		4	
**Total mass in all injections [µg]**	2.84	13.88	12.56
**Volume/fraction [mL][1]**	3.5	3	5
**Total volume of fractions [mL]**	14	12	20
**Aliquot shipped to laboratories [mL]**		5	
**Absolute theoretical mass in shipped 5 mL [µg][2]**	1.02	5.78	3.13

[1] Volume of collected fraction of single injection
[2] Assuming fraction recovery of 100 %

Table S6: Instrumental settings for pyGC-MS analysis.

	Laboratory 1	Laboratory 2
	**Pyrolyzer**	
**Pyrolyzer**	Frontier Multi-Shot EGA/PY-3030D microfurnace pyrolyser (Frontier Laboratories Ltd., Japan)	Frontier Multi-Shot EGA/PY-3030D microfurnace pyrolyser (Frontier Laboratories Ltd., Japan)
**Pyrolyzer Furnace temperature [°C]**	550	600
**Pyrolyzer Interface temperature [°C]**	280	320
**Operation mode**	Single shot	Single shot
	**GC-MS System**	
**GC-MS system**	Agilent 8890/5977B (Agilent Technologies, USA)	Agilent 7890A/ 5975C (Agilent Technologies, USA)
**Injector type**	Split/splitless	Split/splitless
**Injector temperature [°C]**	280	320
**Split ratio**	20:1	25:1
**Oven program**	40 °C (5 min) and ramped up by 20 °C min^{-1} up until it reached 315 °C, 5 min hold, total runtime: 23.75 min	40 °C (2 min) and ramped up by 20 °C min^{-1} until it reached 320 °C, 25 min hold, total runtime: 43 min
**Column Type**	HP-5MS ultra inert-capillary column (Agilent Technologies, 30 m column length, 0.25 mm I.D., 1 µm film thickness)	Frontier Ultra ALLOY+-5 capillary column (30 m length, 0.25 µm film thickness, and 0.25 mm internal diameter)
**Carrier gas**	Helium	Helium
**Flow**	1 mL min^{-1} (constant flow)	1 mL min^{-1} (constant flow)
**Transfer line temperature [°C]**	310	320
**Ion source temperature [°C]**	230	230
**Quadrupole temperature [°C]**	150	150
**Acquisition Mode**	Total ion mode	Selected ion mode
**Calibration**	External	External
**Marker compound indicator ion**	2,4-diphenyl-1-butene, m/z 91 (Styrene dimer)	Styrene monomer (104, 78 m/z), dimer (91, 208 m/z) and trimer (91, 312 m/z). Quantification with monomer.

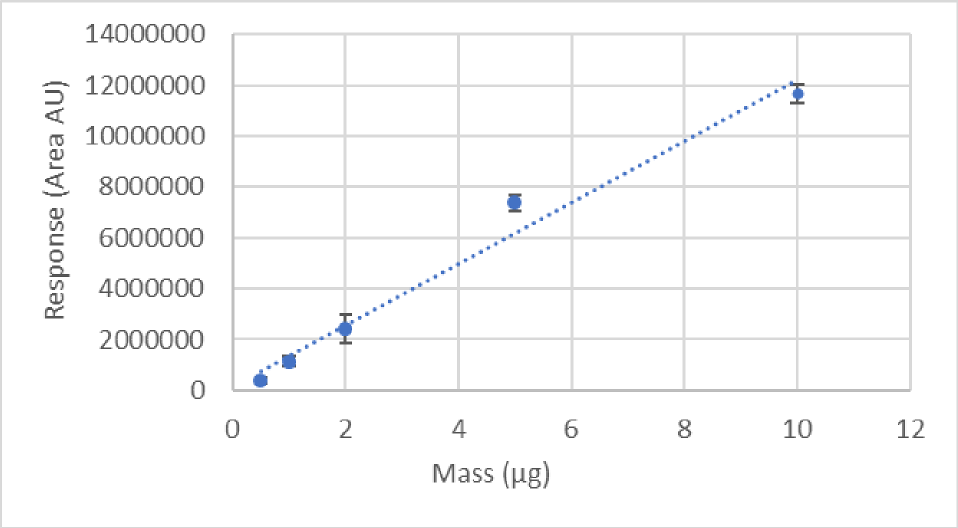

Figure S2: Calibration curve for offline AF4-pyGC-MS measurements of NPLs.

Supplementary figures and tables for the AF4-pyGC-MS analyses

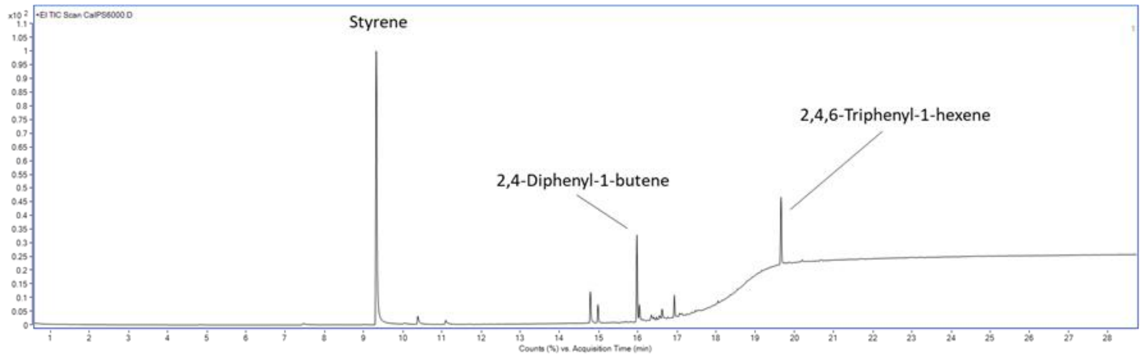

Figure S3: Pyrogram of polystyrene with the three characteristic compounds corresponding to the styrene monomer, dimer (2,4-diphenyl-1-butene) and trimer (2,4,6-triphenyl-1-hexene).

Calibration for PS analysis with pyGC-MS

Based on the expected absolute theoretical mass of polystyrene in each of the collected AF4-fractions, a calibration curve was created ranging from 0.5 to 10 µg (table S7). To this end, solid polystyrene from the "Hawai'i Pacific University Polymer kit 1.0 (PS-HW)" was weighed into 20 mL crimp-top glass vials and 10 mL of ethylacetate were added. The vials were then capped and placed on an orbital shaker overnight at room temperature. Completeness of dissolution was verified by visual inspection. Volumes of 20 µL were then transferred to the pyrolysis cups (volume of cup: 50 µL) and the solvent evaporated overnight at 40 °C.

Table S7: Calibration solutions for pyGC-MS.

Calibration level	Weighed mass of polystyrene [mg]	Volume in which polystyrene was dissolved [mL]	Concentration [µg mL^{-1}]	Mass polystyrene in aliquot (20µL) transferred to pyrolysis cup [µg]
1	0.25	10	25	0.5
2	0.5	10	50	1
3	1	10	100	2
4	2.5	10	250	5
5	5	10	500	10

In absence of an auto sampling system, complete sets of calibration samples for AF4-pyGC-MS were run only on the first, the central and the final day of analysis. The calibration curves generated on three different days matched well (Figure S2), indicating a stable response over the entire period of analysis. An instrument calibration was performed for a mass range of 0.5 -10 µg. The low standard deviations indicate great stability over the entire period of measurements. Response-stability was verified on a daily basis by pyrolyzing in triplicate the 2 µg polystyrene sample only. The relative standard deviation of the responses of all control samples ranged from 3.5% to 7.3%.

Comparison of DLS data from two laboratories

Table S8: Detailed DLS data sets of two laboratories reporting the average hydrodynamic diameter, dispersity and the respective standard deviations.

Sample	d_{DLS1} Z-average [nm] (Dispersity) ([-])	d_{DLS1} SD (nm) (Dispersity (-))	d_{DLS2} Z-average [nm] (Dispersity) ([-])	d_{DLS2} SD (nm) (Dispersity (-))
PE1	911 (0.547)	34 (0.087)	773 (0.478)	70 (0.13)
PS1	140 (0.016)	1 (0.012)	141 (0.001)	1 (-)
PS2	159 (0.019)	1 (0.012)	148(0.001)	2 (-)
PS3	346 (0.163)	3 (0.019)	329 (0.122)	3 (0.023)
FeOx100	187 (0.089)	2 (0.016)	159 (0.056)	0.8 (0.018)
FeOx2000	197 (0.091)	2 (0.022)	310 (0.295)	10 (0.06)

TEM and SEM images

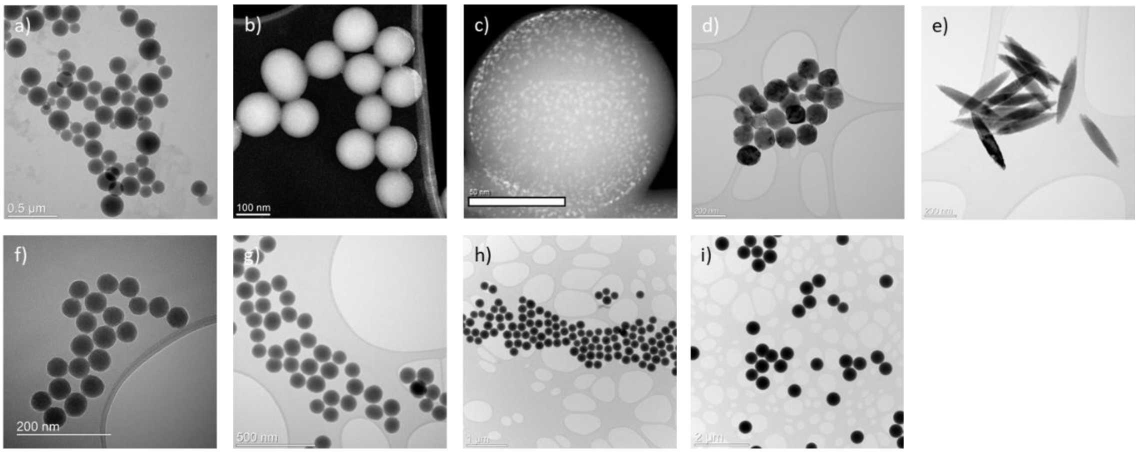

Figure S4: TEM images: a) PE1 (d_{mean} = 145 nm, d_{10} = 68 nm, d_{90} = 205 nm, 51 particles), b) PS2 (d_{mean} = 119 nm, d_{10} = 106 nm, d_{90} = 132 nm, 12 particles), PS2 close-up, d) FeOx100 (d_{mean} = 119 nm, d_{10} = 96 nm, d_{90} = 139 nm, 19 particles), e) FeOx2000 (d_{mean} = 86 nm, aspect ratio = 4.96, 3 particles), f) SISN50 (d_{mean} = 46 nm, d_{10} = 43 nm, d_{90} = 47 nm, 25 particles), g) SISN100 (d_{mean} = 98 nm, d_{10} = 87 nm, d_{90} = 108 nm, 35 particles), h) SISN200 (d_{mean} = 195 nm, d_{10} = 178 nm, d_{90} = 211 nm, 106 particles), i) SISN500 (d_{mean} = 479 nm, d_{10} = 438 nm, d_{90} = 504 nm, 30 particles).

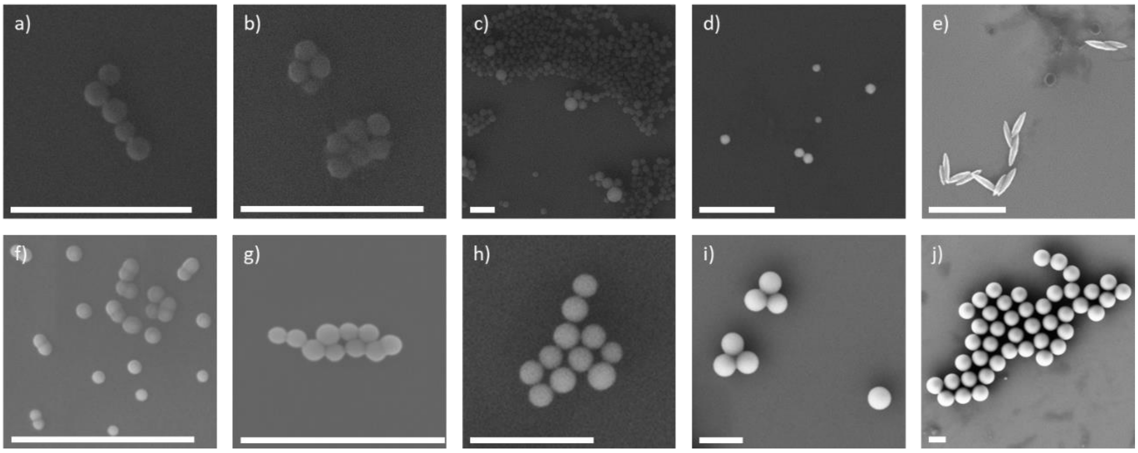

Figure S5: SEM images (white scale bar indicates 1 µm): a) PS1 (103 mg L^{-1}, 15 kV, WD = 7.0 mm, SE), b) PS2 (160 mg L^{-1}, 10 kV, WD = 6.9 mm, SE), c) PS3 (160 mg L^{-1}, 10 kV, WD = 6.9 mm, SE), d) FeOx100 (500 mg L^{-1}, 10 kV, WD = 5.5 mm, SE), e) FeOx2000 (500 mg L^{-1}, 10 kV, WD = 5.7 mm, in-lens), f) SISN50 (150 mg L^{-1}, 3 kV, WD = 4.1 mm, in-lens), g) SISN100 (150 mg L^{-1}, 3 kV, WD = 4.0 mm, in-lens), h) SISN200 (150 mg L^{-1}, 10 kV, WD = 6.8 mm, SE), i) SISN500 (150 mg L^{-1}, 5 kV, WD = 6.3 mm, SE), j) SISN1000 (150 mg L^{-1}, 5 kV, WD = 4.6 mm, SE).

Additional information on CLS measurements

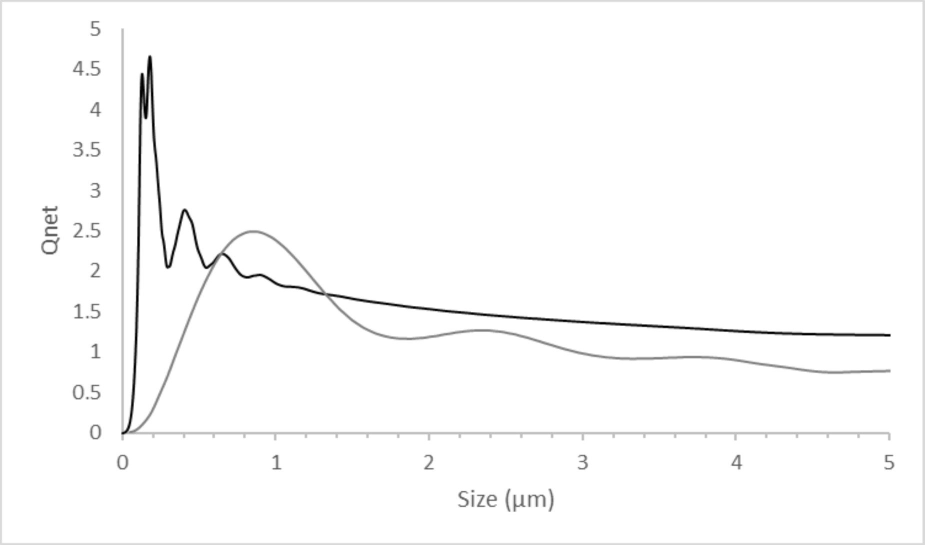

Figure S6: Extinction efficiency functions (Qnet) generated by the software of the disc centrifuge instrument applying Mie theory for spherical iron-oxide (black) and polystyrene (grey) particles.

Additional information on the particle concentrations determined by TRPS, NTA, and CLS

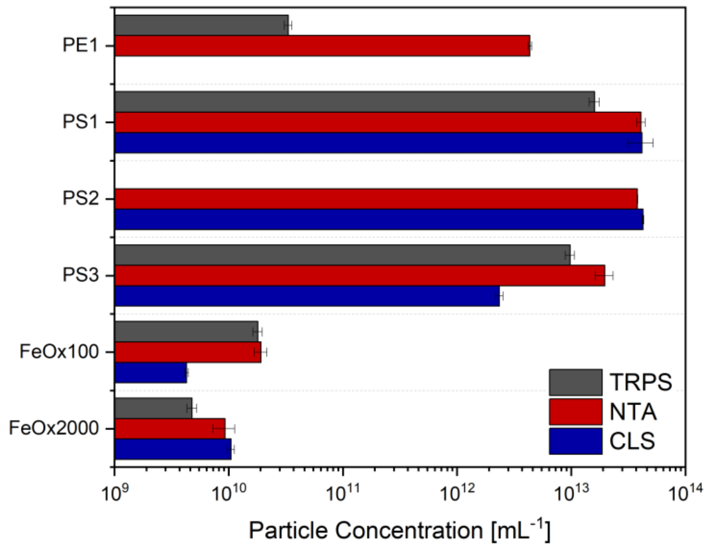

Figure S7: Comparison of the particle concentrations determined by TRPS, NTA, and CLS for the PE, PS and FeOx samples.

Batch Raman microspectroscopy analysis

Batch Raman analysis was performed on the NPL, iron oxide and silica samples to characterize the materials. Most NPL samples showed the expected Raman signatures according to their material (i.e., PS and PE). The Raman spectra of the iron oxide samples revealed the presence of different polymorphs. Both samples showed mainly hematite (α-Fe_2O_3), but in several spectra also a band at 670 cm^{-1}, which is indicative for magnetite (Fe_3O_4), was observed. The presence of magnetite could

indicate incomplete oxidation during production. Furthermore, the Raman analysis of the silica samples suggested an amorphous material due to a broad, weak band around 500 cm^{-1}. The Raman band at 488 cm^{-1} which is indicative of α-quartz is only present in a few spectra.

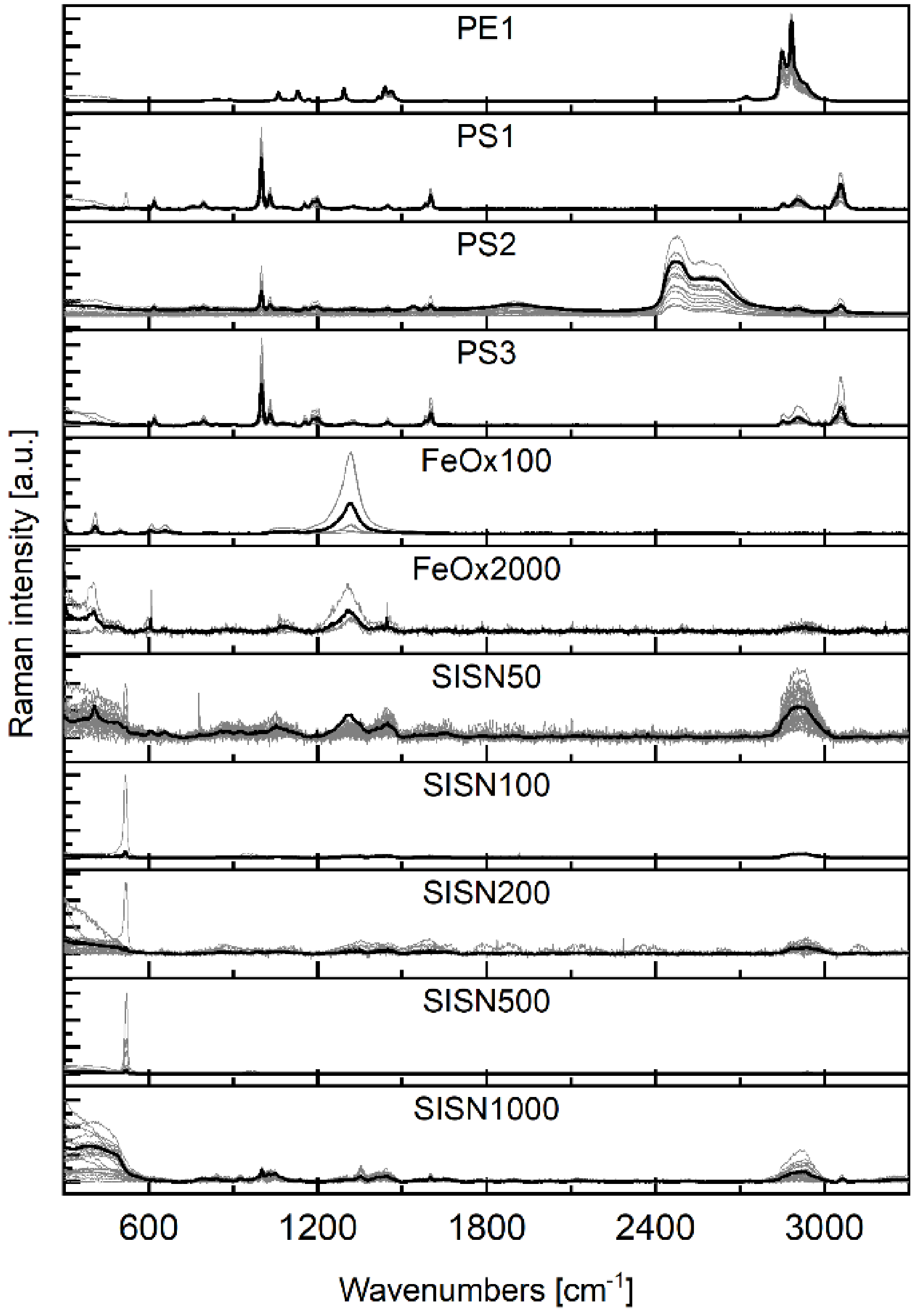

Figure S8: Batch Raman analysis (mean spectra: black, single spectra: grey) of the NPL, iron oxide and silica samples. The Raman bands in the CH-stretch region (around 2900 cm^{-1}) visible in the spectra for most silica samples can be attributed to the surfactant used for dilution.

Dilution factors used for the analysis of particle concentration with NTA, TRPS, and CLS

Table S9: Overview over the techniques used for particle number-based quantification. The stock concentration of the sample is denoted for reference. The particle concentrations measured with TRPS, NTA and CLS are reported including the respective dilution factor. NM=not measured

Sample	Stock concentration [g L^{-1}]	C_{TRPS} Mean [mL^{-1}] (Dilution factor)	C_{NTA} Mean [mL^{-1}] (Dilution factor)	C_{CLS} Mean [mL^{-1}] (Dilution factor)
PE1	50	$3.33*10^{10}$ (700)	$4.34*10^{12}$ (2000)	NM[2]
PS1	103	$1.60*10^{13}$ (5500)	$4.08*10^{13}$ (10^5)	$4.17*10^{13}$ (10)
PS2	160	NM	$3.80*10^{13}$ (10^5)	$4.26*10^{13}$ (10)
PS3	160	$9.77*10^{12}$ (9000)	$1.97*10^{13}$ (10^5)	$2.35*10^{12}$ (10)
FeOx100	Not provided[1]	$1.80*10^{10}$ (30)	$1.92*10^{10}$ (100)	$4.28*10^{9}$ (20)
FeOx2000	Not provided[1]	$4.78*10^{9}$ (30)	$9.28*10^{9}$ (50)	$1.05*10^{10}$ (10)
SISN50	Not provided[1]	$4.02*10^{12}$ (2000)	NM	$1.06*10^{13}$ (10)
SISN100	Not provided[1]	$1.43*10^{13}$ (1000)	$8.07*10^{12}$ (25000)	$2.84*10^{12}$ (10)
SISN200	Not provided[1]	$4.94*10^{11}$ (200)	$2.55*10^{12}$ (10000)	$5.07*10^{11}$ (10)
SISN500	Not provided[1]	$5.18*10^{10}$ (75)	$2.56*10^{11}$ (1000)	$3.78*10^{10}$ (10)
SISN1000	Not provided[1]	$1.01*10^{10}$ (20)	NM	$5.69*10^{9}$ (10)

[1] Mass concentration of the stock not provided.

[2] Not possible to measure in standard setup due to the particles floating.

Comparison of CF3-MALS data from two laboratories

Table S10: Comparison of CF3-MALS data from two laboratories, including retention time (RT), full-width-at-half-maximum (FWHM) and percentage recovery.

Sample	RT [min] (Lab 1)	± SD	RT [min] (Lab 2)	± SD	FWHM [min] (Lab 1)	± SD	FWHM [min] (Lab 2)	± SD	% Recovery (Lab 1)	± SD	% Recovery (Lab 2)	± SD
PE1	64.20	0.09	54.53	0.18	47.11	0.57	31.25	0.39	96.98	3.28	84.48	4.19
PS1	26.79	0.01	43.06	0.26	7.57	0.01	14.77	0.12	100.20	0.07	91.84	1.04
PS2	27.71	0.75	43.83	0.26	7.70	0.03	13.72	0.08	92.91	0.75	86.43	2.17
PS3	44.94	0.39	63.14	0.41	13.38	0.19	15.76	0.06	97.98	0.97	100.87	0.94
FeOx100	49.45	0.06	-	-	16.15	0.36	-	-	95.27	5.48	-	-
FeOx2000	47.85	0.13	-	-	10.65	0.59	-	-	91.55	7.39	-	-

Table S11: Size ranges of different samples obtained from CF3-MALS data.

Sample	R_g (peak max; [nm]) (Lab 1)	± SD	R_g (peak max; [nm]) (Lab 2)	± SD	R_g range[1](min) (Lab 1) [nm]	R_g range[1] (max) (Lab 1) [nm]	R_g range[1] (min) (Lab 2) [nm]	R_g range[1] (max) (Lab 2) [nm]
PE1	664.50	52.50	108.25	0.75	115.0	717[2]	47.8	249.2
PS1	51.90	0.10	56.12	0.21	44.9	63.9	47.0	146.2
PS2	54.50	1.00	57.30	0.16	48.5	70.0	54.0	88.7
PS3	120.33	2.83	115.27	0.61	80.9	262.8	85.4	180.5
FeOx100	77.37	1.13	-	-	66.4	155.8	-	-
FeOx2000	92.77	0.57	-	-	75.7	216.3	-	-

[1] The range was determined by evaluating the radius of gyration (R_g) at 10% intensity of the MALS 90° signal.

[2] This R_g value was determined at the maximum MALS 90° signal since beyond no meaningful results could be obtained due to the upper working range of the MALS detector.
